# Isolation and structure of broad SIV-neutralizing antibodies reveal a proximal helical MPER epitope recognized by a rhesus multi-donor class

**DOI:** 10.1016/j.celrep.2024.115163

**Published:** 2025-01-09

**Authors:** Jason Gorman, Renguang Du, Yen-Ting Lai, Mohammed S. Ahmadi, Hannah A.D. King, Kaimei Song, Kimberly Manalang, Christopher A. Gonelli, Chaim A. Schramm, Cheng Cheng, Richard Nguyen, David Ambrozak, Aliaksandr Druz, Chen-Hsiang Shen, Yongping Yang, Daniel C. Douek, Peter D. Kwong, Mario Roederer, Rosemarie D. Mason

**Affiliations:** 1 Vaccine Research Center, National Institute of Allergy and Infectious Diseases, National Institutes of Health, Bethesda, MD 20892, USA; 2 Division of Viral Products, Center for Biologics Evaluation and Research, Food and Drug Administration, Silver Spring, MD 20993, USA; 3 Aaron Diamond AIDS Research Center, Columbia University Vagelos College of Physicians and Surgeons, New York, NY 10032, USA; 4 Lead contact

## Abstract

The membrane-proximal external region (MPER) of the HIV-1 envelope is a target for broadly neutralizing antibodies (bnAbs), and vaccine-elicited MPER-directed antibodies have recently been reported from a human clinical trial. In this study, we sought to identify MPER-directed nAbs in simian immunodeficiency virus (SIV)-infected rhesus macaques. We isolated four lineages of SIV MPER-directed nAbs from two SIV-infected macaques. The nAbs displayed low potency but up to 90% breadth on a 20-strain SIV panel. Crystal structures of representative nAbs in complex with SIV MPER peptides revealed the SIV antibodies to bind a helical epitope at the N-terminal (proximal) region of the MPER, defining a reproducible multi-donor class encompassing all four lineages. HIV-1 comparison showed that this class of SIV MPER-directed antibodies targets a helical region overlapping that targeted by human vaccine-elicited ones. Thus, a prevalent and reproducible class of SIV bnAbs recognizes an epitope similar to that recently observed in an HIV-1-vaccine trial.

## In brief

The membrane-proximal external region (MPER) of the HIV-1 envelope is a target for broadly neutralizing antibodies (nAbs) and vaccine development. Gorman et al. describe four SIV MPER-directed antibody lineages, from two rhesus macaques, that share immunogenetics and bind the MPER in a similar manner, thereby comprising a reproducible class of SIV bnAbs.

## Graphical Abstract

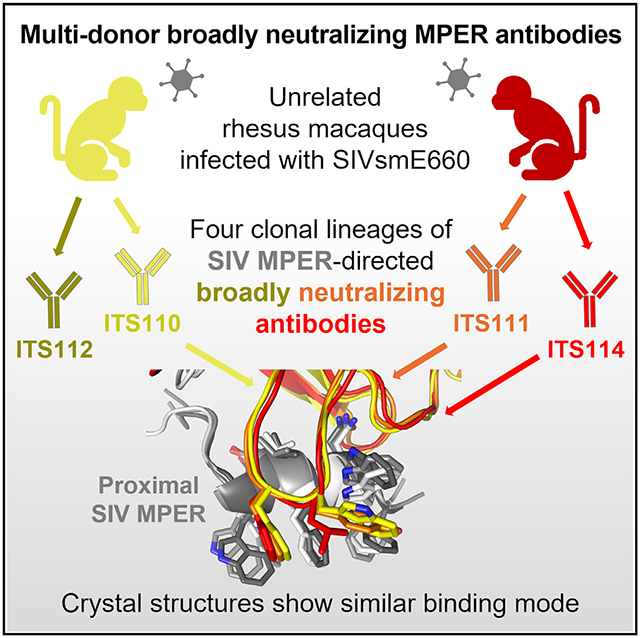

## INTRODUCTION

The simian immunodeficiency virus (SIV) and simian human immunodeficiency virus (SHIV) non-human primate (NHP) models have contributed substantially to our understanding of human immunodeficiency virus (HIV)-1 disease pathogenesis, treatment options, and vaccine candidates. Until recently, both models were limited in their utility owing largely to the lack of clinically relevant pathogenic SHIV strains and the paucity of rhesus-derived HIV-/SIV-specific monoclonal antibodies (mAbs) from SHIV-/SIV-infected rhesus macaques. However, there is now a range of well-characterized SHIV strains incorporating more clinically relevant HIV-1 envelope (Env) transmitted founder virus sequences well suited to vaccine studies.^1–5^ Likewise, several HIV-/SIV-specific mAbs have been isolated from rhesus macaques infected with SHIV or SIV, respectively, including several neutralizing antibodies (nAbs) analogous to HIV-1 broadly nAb (bnAb) candidates being evaluated for clinical use in the treatment and prevention of HIV-1.^6–9^

We have previously reported on the isolation and characterization of multiple SIV-specific mAbs targeting various epitopes on SIV Env.^6,7,9^ These include SIV mAbs targeting sites corresponding to common HIV-1 Env bnAb epitopes, such as the CD4-binding site (CD4bs), the CD4-induced (CD4i) site, variable loops 1, 2, and 3 (V1, V2, V3), the high-mannose patch, and the interface region of SIV Env. However, SIV mAbs specific for the membrane-proximal external region (MPER), a site targeted by some of the broadest and most potent HIV-1 bnAbs,^10–17^ have yet to be isolated. The conservation of the MPER and corresponding breadth of bnAbs targeting it make it an attractive epitope for vaccination. Indeed, a recent clinical trial elicited tier 2 nAb responses through MPER-peptide liposome immunizations.^18^ Here, we describe the isolation and characterization of 13 SIV MPER-specific mAbs from two rhesus macaques. Antibody repertoire analysis combined with crystal structure analysis of three mAbs in complex with SIV MPER peptide suggest that these 13 mAbs belong to four clonal families comprising a singular multi-donor antibody class, indicating reproducible SIV MPER mAb development. These SIV MPER mAbs expand the SIV mAb toolkit being used to evaluate and screen HIV-1 mAb-based intervention strategies.^19,20^ More importantly, these SIV MPER mAbs recognize a helical epitope similar to recently isolated antibodies from a human clinical trial.^18^

## RESULTS

### Isolation of MPER-specific mAbs from SIV-infected rhesus macaques

**Figure 1.**
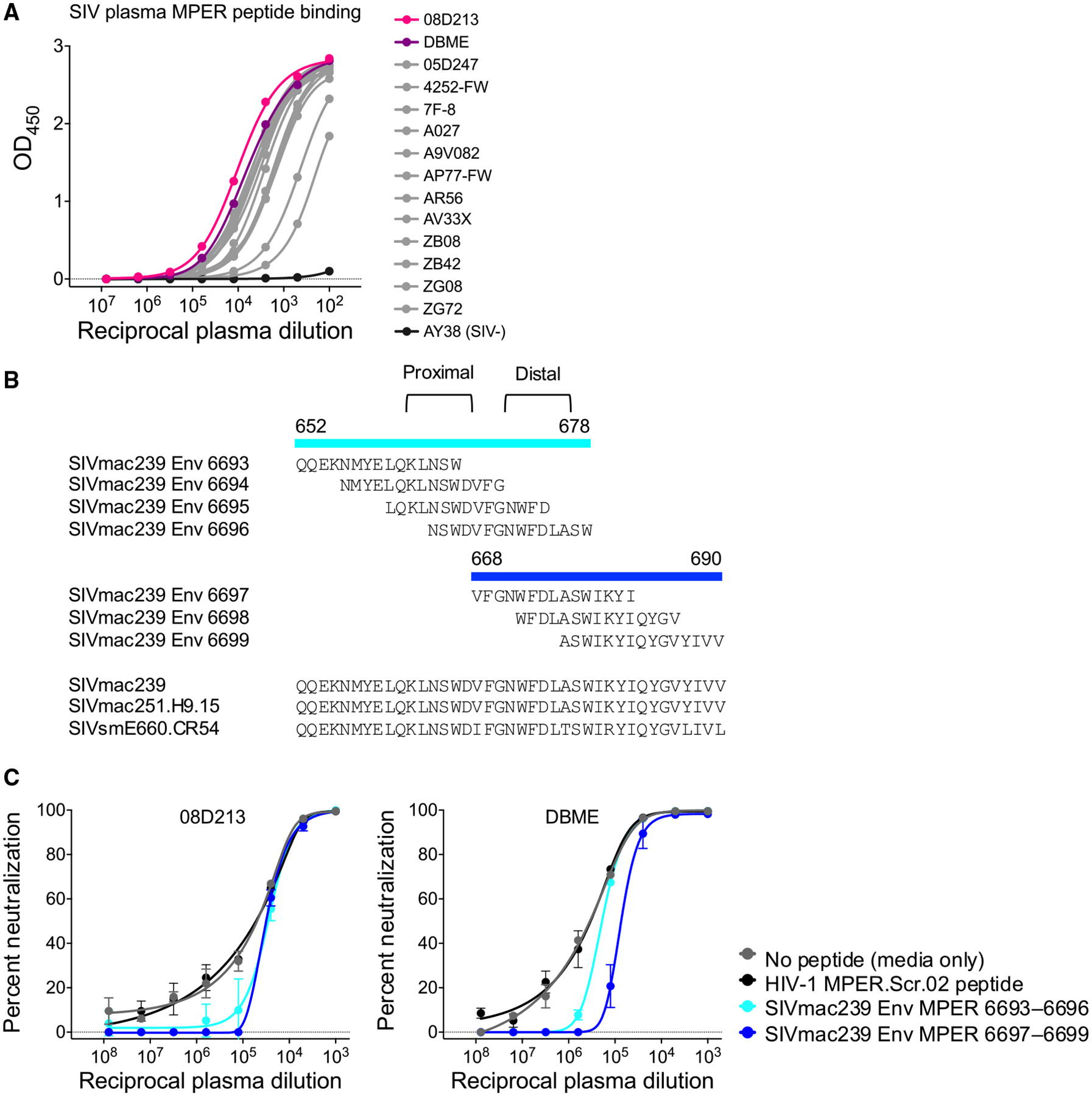
SIV MPER-directed neutralization detected in SIVsmE660-infected rhesus macaques (A) ELISA binding activity of SIV+ and SIV− rhesus plasma to a pool of overlapping peptides spanning SIVmac239 MPER (SIVmac239 Env 6693–6699). (B) Definition of MPER peptide sequences used in (A) and (B). Core binding regions of proximal and distal HIV-1 MPER antibodies are shown. SIVmac239, SIVmac251.H9.15, and SIVmacE660 have nearly identical sequences for this region. (C) Neutralizing activity of SIVmac251.H9.15 (tier 1) by SIV+ rhesus plasma samples in the presence or absence of peptides spanning SIVmac239 MPER or control HIV-1 MPER scrambled peptide (HIV-1 MPER.Scr.02). The detection limit is indicated by a horizontal dashed line. Plotted mean and standard deviation (SD) error bars shown.

**Figure 2.**
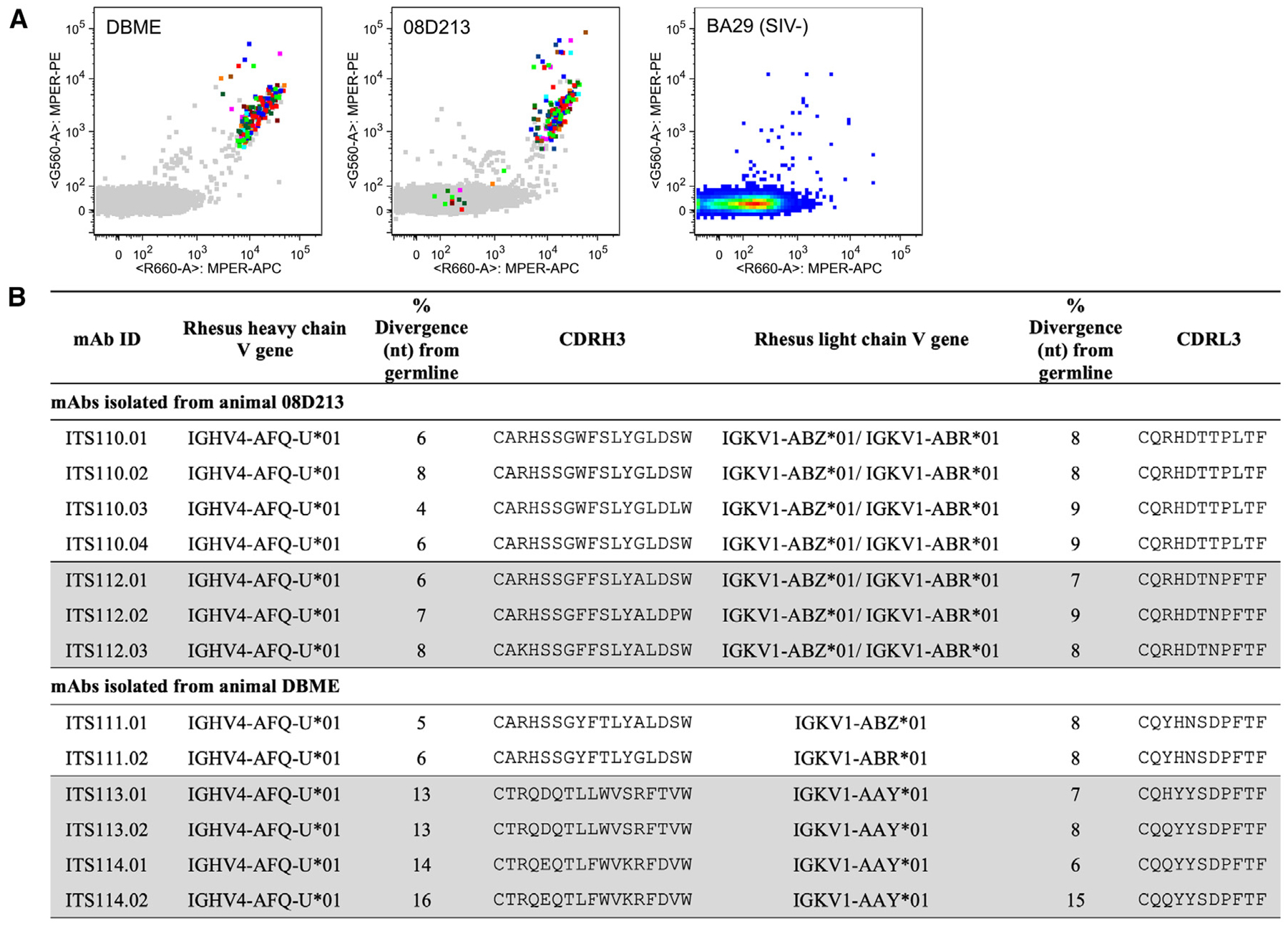
Isolation and sequencing of SIV MPER-directed mAbs (A) FACS plots of CD20+IgM− IgG+ memory B cells from SIV+ rhesus macaques (left and middle) showing dual SIV MPER peptide-binding (i.e., SIV MPER-APC+ and SIV MPER-PE+) B cells (multicolored) overlayed on total memory B cells (gray). SIV MPER peptide staining of memory B cells from a SIV-uninfected rhesus macaque (right) is shown for comparison. (B) Immunogenetics of 4 lineages of SIV MPER neutralizing mAbs isolated from animals 08D213 and DBME.

Several HIV-1 MPER bnAbs have been isolated by fluorescence-activated cell sorting (FACS) using fluorescently labeled MPER peptides^13,21^; therefore, we screened plasma from 14 SIV-infected rhesus macaques by ELISA for binding to a pool of 15-mer peptides spanning the SIV MPER. All SIV-infected animals tested showed binding to the SIV MPER peptide pool, with 08D213 and DBME displaying some of the strongest signals, while a control SIV-uninfected animal was negative for binding to the same peptide pool (Figures 1A and 1B). To determine whether neutralizing activity in animals 08D213 and DBME mapped to the MPER, we tested whether neutralization of SIVmac251 virus could be blocked by the addition of soluble SIV MPER peptides. SIV MPER peptides, but not the blank control or the scrambled HIV-1 MPER peptide (MPER.Scr.02), partially blocked plasma neutralization of SIVmac251 in 2 rhesus macaques (Figure 1C). This suggested the possibility of using fluorescently labeled tetramers of the SIV MPER peptide to label and sort SIV MPER-specific B cells from peripheral blood mononuclear cells (PBMCs) matched to the plasma time points used for screening by ELISA binding to SIV MPER peptides from these two animals. Since peptide probes often yield high background binding, we used dual SIV MPER probe staining to sort and gate dual SIV MPER peptide-binding B cells from animals 08D213 and DBME (Figure 2A). Of the 1–1.5 million PBMCs acquired, approximately 0.3%–0.4% of memory B cells were dual positive for SIV MPER peptide binding, similar to the frequency of MPER-specific B cells observed in HIV-infected donors.^13,21^

**Figure 3.**
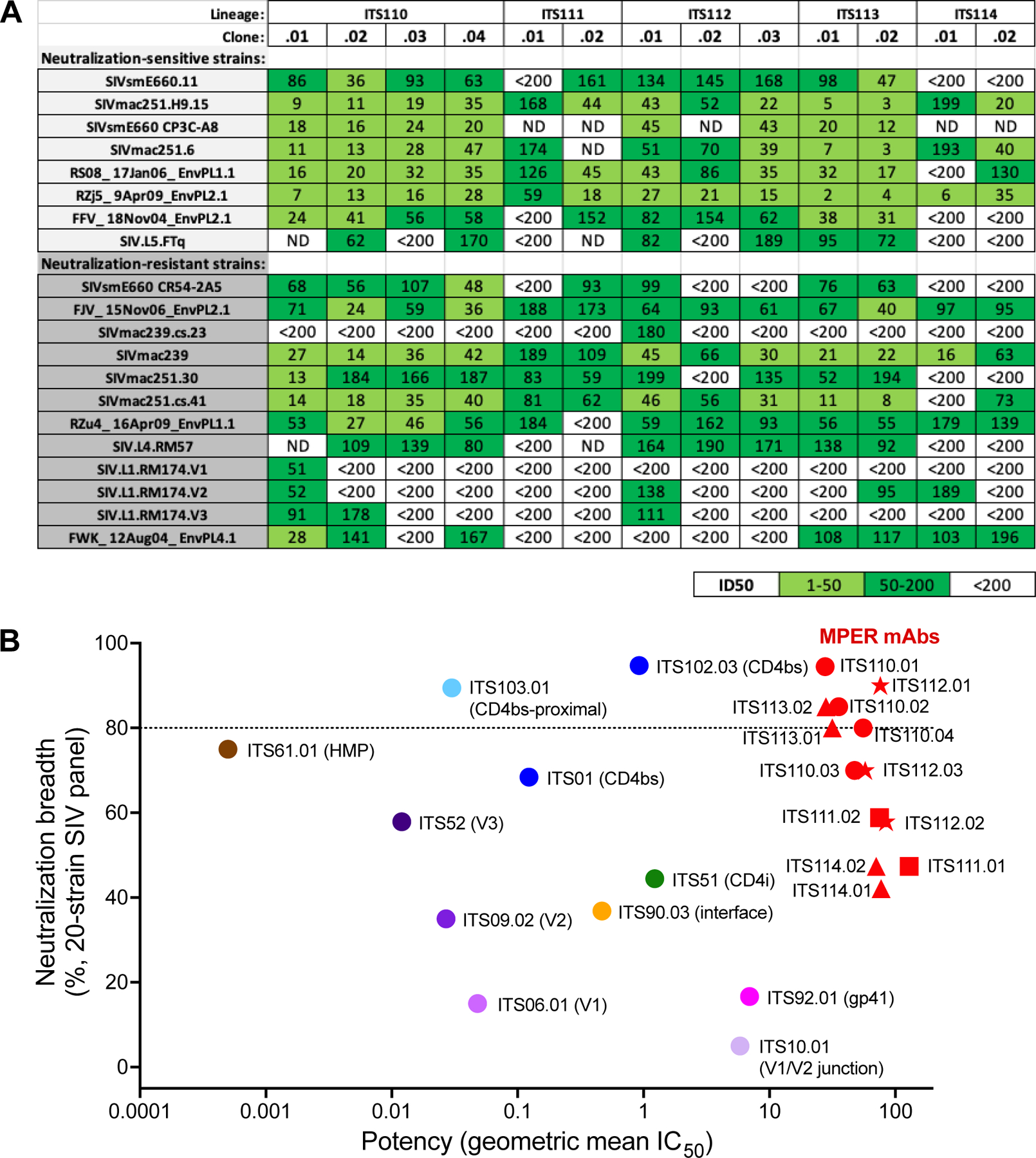
Breadth and potency of SIV MPER-directed Nabs (A) Neutralization of SIV pseudo-typed virus panel by SIV MPER nAbs isolated from animals 08D213 and DBME. See also Figures S1–S3. ND, not determined. (B) Breadth potency plot showing breadth as percentage of SIV isolates tested and neutralized at an IC_50_ <50 μg/mL (non-MPER-directed) or <200 μg/mL (MPER-directed) in pseudo-typed virus neutralization assays plotted against the geometric mean inhibitory concentration (IC_50_ in μg/mL) for all neutralization-sensitive viruses (potency). MPER-directed mAbs are shown in red symbols with different symbols for each lineage (circle for ITS110; square for ITS111; star for ITS112; triangle for ITS113 or ITS114).

We expressed 21 and 26 mAbs from animals 08D213 and DBME, respectively, and screened them for neutralizing activity against the highly neutralization-sensitive strains SIVsmE660.11 and SIVmac251.H9.15. A total of 13 mAbs (7 from 08D213 and 6 from DBME) were positive for neutralization and down-selected for assessing neutralization against our panel of 20 SIV strains, which closely reflects inter-clade genetic diversity of HIV-1 and includes both neutralization-sensitive and -resistant SIV strains^6,9^ (Figures 2B and 3A). Analysis of the immunoglobulin (Ig) heavy- and light-chain nucleotide sequences of SIV MPER-specific neutralizing mAbs revealed that the 7 mAbs from 08D213 belonged to 2 clonal families (designated ITS110 and ITS112) based on the distinct heavy-chain third complementarity determining region (CDRH3) N2 insertions and different light-chain J genes (Figures 2B and S1). The 6 mAbs from DBME also belonged to 2 clonal lineages, with the first being ITS111 and the second divided into ITS113 and ITS114 (manual/expert junction analysis was used to conclude that ITS113 and ITS114 are likely the same lineage), with all appearing to use the same putative germline gene sequences (Figures 2B and S1). Due to the similarity in B cell lineages isolated from different animals, phylogenetic analysis of concatenated heavy- and light-chain sequences was performed to rule of the possibility of cross-contamination (Figure S5 and Table S3). ITS113 and ITS114 are most likely the same lineage but are assigned their own ITS code since there is enough uncertainty in the D gene assignment that it is possible that they are in fact two lineages (Figure S1). Clonal members of ITS110, ITS111, and ITS112 lineage mAbs were less divergent (4%–8% for variable heavy [VH] chain; 7%–9% for variable kappa (Vκ) chain from their putative germline gene sequences than those of ITS113 and ITS114 mAbs (13%–16% for VH; 6%–15% for Vκ).

Although the breadth of neutralization of SIV MPER-specific mAbs was greater than that observed for other SIV mAb classes, the responses were of low potency (Figures 3A and 3B). We observed no neutralization plateaus, as has been seen with several SIV antibodies^6^ (Figure S2). To determine whether the Fc portion of these mAbs was limiting access to the SIV MPER and hence affecting potency, we used pepsin to cleave mAbs in the lower hinge/CH2 region to generate F(ab′)2 fragments of mAbs from each lineage to assess virus neutralization. Compared to intact IgG, there was no increase in neutralization potency of SIV by F(ab′)2 fragments, indicating that the low potency of SIV MPER mAbs was not due to steric hinderance of the Fc region (Figure S2).

### Crystal structures reveal similar modes of binding of ITS110, ITS111, and ITS114

**Figure 4.**
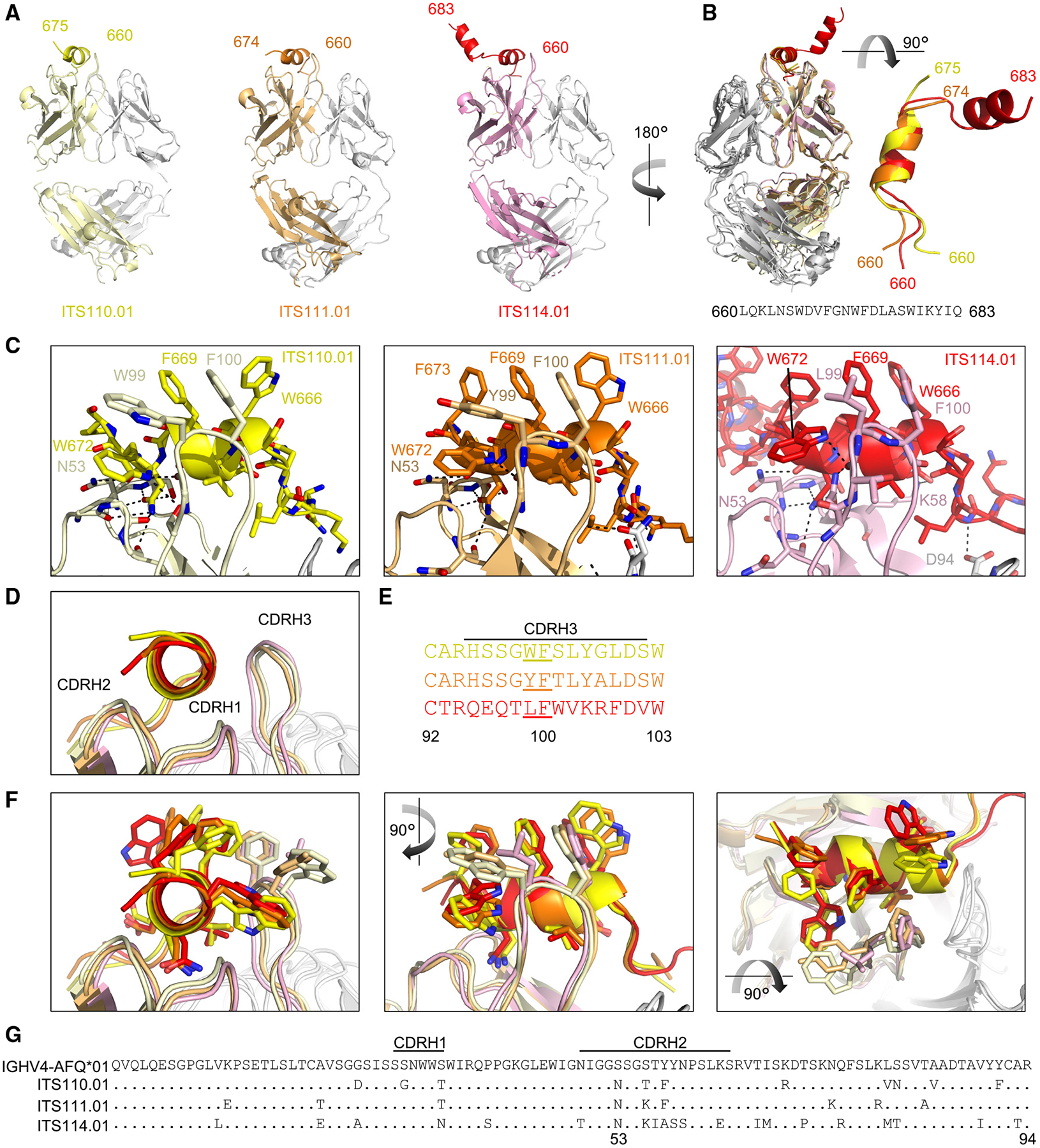
Crystal structures reveal similar MPER binding of ITS110.01, ITS111.01, and ITS114.01 (A) Overall structures of ITS110.01, ITS111.01, and ITS114.01 are shown in cartoon format, aligned by heavy chains. (B) Overlay of the alignment of the three antibodies with peptide (left) and the peptide alone from the same alignment rotated 90°. Density for the C termini of the peptides that did not allow for accurate fitting was not modeled. (C) H-bonds and hydrophobic interactions are shown for each of the three antibodies. (D) An overlay is shown of the antibodies aligned by the heavy chain. MPER peptides beyond 673 are not shown. (E) An alignment of the CDRH3 of the three antibodies are shown, with residues 99 and 100 underlined. (F) Overlay as in (D), showing side chains from difference angles. (G) Sequence alignment of the heavy V-gene and maturation mutations for each antibody is shown. See also Figure S3.

To investigate the structural basis for recognition of the SIV MPER epitope by ITS110, ITS111, and ITS114 antibodies, we determined the crystal structures of each antibody in complex with the SIV MPER peptide at resolutions of 3.6, 2.0, and 3.0 Å, respectively (Figure 4A; Table 1). The final refined models exhibited R-work and R-free values of 0.26, 0.24, and 0.26 and 0.28, 0.26, and 0.30, respectively. The SIV MPER peptide, referred to as SIV_mac239_-MPER-3R, included residues 660–683 with three Arg residues at the C terminus to increase solubility. Density for each structure was clear for peptide residues 661–673; however, while some density for the C-terminal region of the peptides beyond residue 674 was observed in all three structures, it was not suitable for accurate modeling in the ITS110 and ITS111 structures. All three antibodies showed a similar mode of binding to the SIV MPER epitope, with their CDRH3, CDRH2, and CDRL3 loops forming a binding pocket that accommodates the MPER peptide (Figures 4A–4F). The overall structure of each antibody-epitope complex revealed a conserved binding mode, in which the heavy chains of the antibodies interacted with the MPER peptide in a similar manner, recognizing conserved residues of the MPER peptide, while the light chain was minimally involved (Figures 4A–4F and S3). In all three structures, the MPER peptide adopted a helical conformation with a conserved cap through a hydrogen bond with N53 of the heavy chain breaking continuation of the helix. The helical region of the MPER is nestled into the binding pocket formed by the CDR loops (Figure 4D), adjoining hydrophobic residues 99 and 100 of the heavy chain, which maintained a conserved loop position in the three structures (Figures 4C–4F).

A shared affinity maturation at position 53 from a germline Ser to a mature Asn was observed to cap the helix in each structure (Figures 4C and 4G). While this S53N mutation is not predicted to be rare by ARMADiLLO analysis,^22^ it was the only mutation from the germline that was found in all members of all lineages. Hydro-phobic residues 99 and 100 near the CDRH3 tips are in close proximity to hydrophobic MPER residues W666 and F669 for the three structures; however, analysis of the buried surface area (BSA) for the MPER revealed residues N671 and W672 to be among those residues with the most surface area contributing to the interface (Figure S3). Although we were not able to produce diffraction-quality crystals for ITS112 lineage members, sequence similarity strongly suggests a mode of binding similar to that observed with other members of this class. Thus, all antibodies from the four lineages bind the MPER in a helical conformation through a common mode of binding.

### ITS110, ITS111, and ITS114 recognize a proximal MPER epitope overlapping multiple human antibodies, including those elicited by vaccination

**Figure 5.**
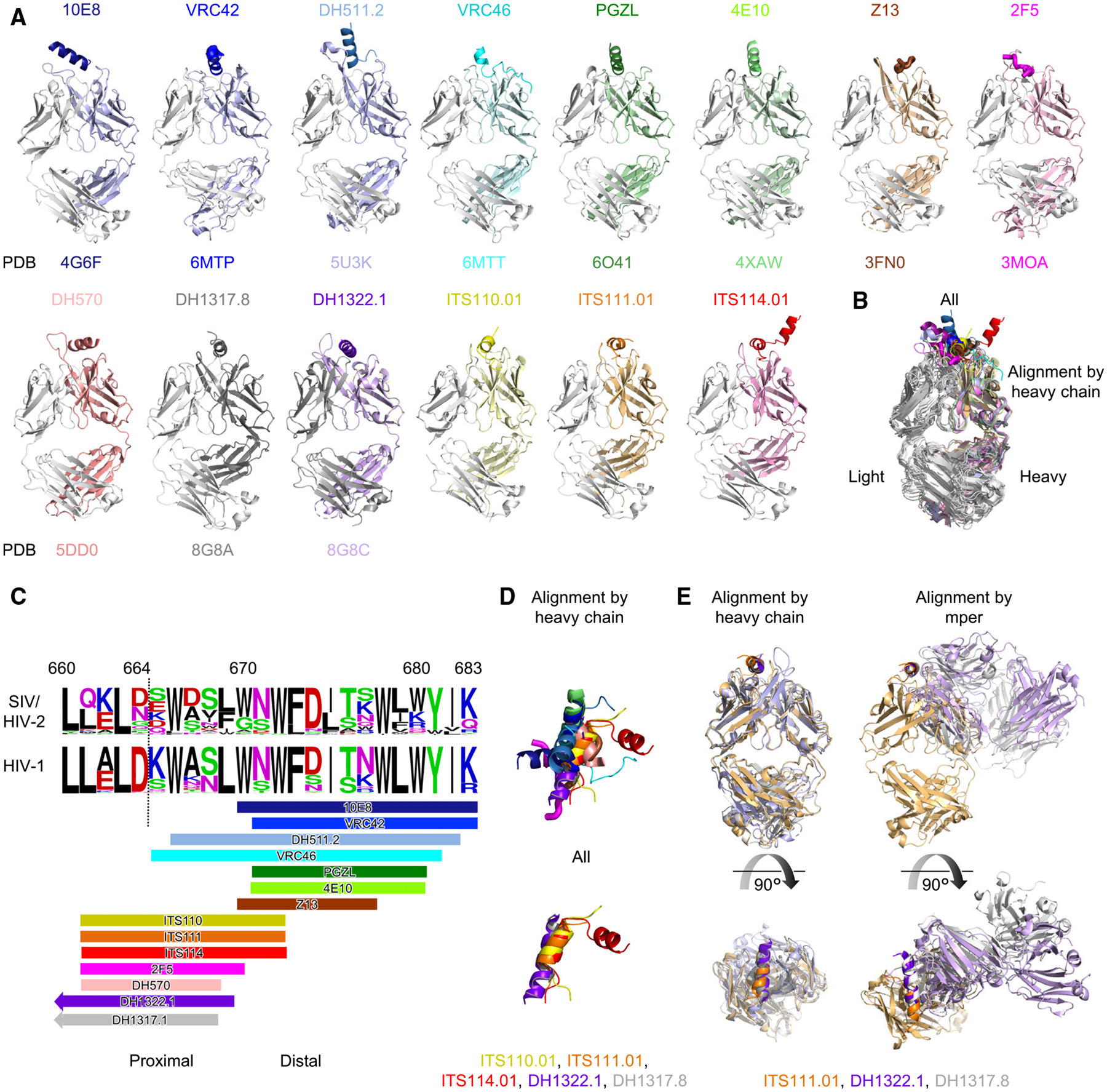
Broad HIV-1 MPER-directed antibodies bind the MPER toward the C-terminal compared to SIV MPER antibodies, and the structure of the MPER is flexible (A) HIV-1 MPER antibodies are shown to compare with the three SIV antibodies, all aligned by the heavy chain. Peptides with no secondary structure are displayed with a larger tube radius for clarity. Some peptides were truncated at flexible turns to only include regions interacting with the antibody. (B) An overlay of all antibodies is shown with alignment by the heavy chain. (C) A logo plot shows the MPER of SIV and HIV-1 from residues 660–683. Binding regions for each of the antibodies in (A) are shown on the bottom. Dashed line delineates the C-terminal of SOSIP.664 constructs. (D) (Top) An overlap of the peptides bound in all of the structures when they are aligned by the heavy chain of each Fab. Orientation is rotated 90° from (B). (Bottom) The closely aligned SIV MPER Fabs with DH1322.1. (E) (Left) DH1322.1 is shown with ITS111.01 aligned by the heavy chain. (Right) DH1322.1 is shown with ITS111.01 aligned by MPER. See also Figures S3 and S4.

Comparative analysis with the crystal structures of HIV-1 MPER antibodies revealed significant differences in the binding mode for all except those of DH1317.8 and DH1322.1, elicited in HVTN 133 (Figure 5A).^18^ The regions bound by 10E8,^12^ 4E10,^23^ VRC42,^14^ and others^13,16,24,25^ begin following the turn after the helix capped by residue Asn53 (Figures 5A–5C). These published HIV-1 MPER epitopes, typical of distal bnAbs, are located down-stream of the binding of ITS110, ITS111, and ITS114 antibodies, which instead overlap with the epitopes of proximal human bnAb 2F5 and rhesus macaque bnAb DH570, although in alternate conformations (Figures 5A–5D). Several human nAbs elicited by vaccination bind to a similar helical epitope that overlaps these SIV bnAbs.^18^ Notably, the binding pocket formed by the CDR loops of the ITS110, ITS111, and ITS114 antibodies accommodated a conformation of the SIV MPER peptide shared by several of these human nAbs, including DH1317.8 and DH1322.1 (Figures 5C–5E). Analysis of the BSA for MPER residues from SIV and HIV-1 shows these antibodies to have substantial overlap in which residues are buried in the interface. Residue W666 is not buried by any helical binders, indicating it may be inaccessible on the full-length Envs of HIV-1 or SIV in the helical conformation, while 2F5 interacts strongly with this residue in a nonstructured conformation (Figure S3). Given that both sets of antibodies are capable of neutralization, this further suggests that this stretch of the MPER can adopt multiple conformations on virions. Interestingly, an ITS113.01 anti-idiotype antibody shows low levels of binding by 2F5 (Figure S4), which may indicate some recapitulation, although details of the anti-idiotypic binding remain unknown. Overall, the crystal structures of the ITS110, ITS111, and ITS114 antibodies in complex with the SIV MPER peptide revealed a helical binding mode at the interface of heavy and light chains, reminiscent of human bnAbs recently isolated in a human clinical trial, although shifted in register.

## DISCUSSION

MPER-specific antibodies contribute significantly to anti-HIV-1 nAb responses. HIV-1 neutralization breadth and potency is substantially increased by the presence of HIV-1 MPER-specific antibodies in sera^26^ and antibody cocktails tested *in vitro*,^27^ and the well-characterized HIV-1 MPER-specific antibodies 4E10 and 10E8 offer potent *in vivo* protection from SHIV infection.^28,29^ Although HIV-1 MPER-directed bnAbs exhibit lower overall potency, they show outstanding breadth, often neutralizing 95%–99% of viruses,^14,16^ including many neutralization-resistant isolates.^27^ Human bnAbs targeting the MPER have arisen in two multi-donor classes,^13,14,16,30^ suggesting multiple reproducible paths to eliciting these types of antibodies. Here, we report the isolation of 13 MPER-directed SIV mAbs from 4 clonal lineages, which likewise exhibit lower overall potency but up to 90% neutralization breadth against a panel of 20 SIV viruses, including many neutralization-resistant isolates,^9^ and target an epitope overlapping that of HIV-1 bnAb 2F5^31^ and vaccine-elicited MPER antibodies.^18^

Strikingly, all three crystal structures show essentially identical binding modes despite arising in three separate lineages and from two different animals. The binding pocket of each antibody overlays closely with CDRH3s that diverge slightly but retain their overall fold and hydrophobic contacts, particularly in residues 99 and 100, which abut the MPER helix. The MPER peptide used in each case adopts the same helical MPER-proximal conformation, which has been recently observed in nAbs elicited in a human clinical trial. All three antibodies also bind a similar overlapping region encompassing residues 661–672. The corresponding region in HIV-1 is bound by 2F5 and DH1317, whereas the majority of broadly neutralizing HIV-1 MPER antibodies bind the distal C-terminal part of the MPER (residues 670–683). Overall, the structures demonstrate a reproducible class of broadly neutralizing SIV MPER antibodies that recognize a proximal helical region similar to those elicited in a human clinical trial.

The MPER epitope targeted by the bnAbs described here suggests that the proximal region of MPER is a potential vaccine target. Most human bnAbs elicited through natural infection target the distal region and do not focus on important sequence components of the proximal region. Vaccination strategies using the soluble HIV-1 Env trimer often terminate at residue 664, primarily for historical reasons; however, by extending just 7 additional residues, an additional broadly neutralizing epitope could be included. For distal MPER antibodies as well as 2F5, membrane interactions are often important for binding; we do not yet know if this is true for the proximal SIV MPER antibodies. Distal binding bnAbs also must contend with the accessibility of the MPER, which may necessitate tilting of the Env ectodomain. For membrane-bound antigens, trimers, or peptides, consideration should be given to the composition of the distal sequence to ensure potential engagement by antibodies analogous to those presented here or in the clinical trial.^18^

Our previous work has led to the isolation and characterization of a variety of SIV-specific rhesus-derived mAbs targeting SIV Env at sites analogous to key sites of vulnerability on the HIV-1 Env, including the CDbs, variable loops, high-mannose patch, and interface region.^6,7,9^ However, only ITS102 and ITS103 bnAbs, which target the CD4bs and a site proximal to the CD4bs, respectively, demonstrate near complete neutralization breadth against our panel of SIV viruses. The isolation of SIV MPER-specific bnAbs described herein expands the range of mAb reagents targeting SIV Env epitopes of interest. In this case, the SIV antibodies have near complete breadth of neutralization against our SIV panel, albeit at lower potency, and bind a conformational target observed in the HIV-1 HVTN133 trial. Overall, the similarity of the MPER-specific bnAbs reported in this study and those from HVTN 133 provide a means to study the elicitation of such antibodies in a model system (NHPs) that is amendable to challenge.

### Limitations of the study

This study utilized a 20-virus panel to assess the breadth of neutralizing activity. While this is a reasonable sample size and was varied to capture as much sequence diversity as possible, it may not represent the full range of SIV strains globally. The nAbs reported here have generally lower potency, which could limit their therapeutic and prophylactic potential unless methods to enhance their activity are explored; however, we feel that the breadth and mechanism are indicative of important antibodies. We also note that the antibodies here showed rising neutralization at typical upper concentration limits and so were tested up to 200 μg/mL. This is higher than many antibodies are screened and should be taken into account when comparing the breadth of these antibodies to others. The crystal structures show that the MPER peptide contacts, given the unknown orientation of such a peptide in the membrane-bound native trimer context, may include additional contacts not seen with the peptide alone. Likewise, without a trimer context, the native accessibility of the epitope is not directly known, nor is the orientation of the antibody with respect to the trimer and membrane. We also acknowledge that there may be a membrane-binding component of the antibodies that we did not explore here. Despite the aforementioned limitations, the high degree of reproducibility observed within this class of nAbs and their similarity to those elicited in HVTN 133 underscore their significance as a promising avenue for future research in HIV-1 nAb-based interventions and vaccine development.

## RESOURCE AVAILABILITY

### Lead contact

Requests for further information should be directed to and will be responded to by rosemarie.mason@nih.gov.

### Materials availability

The plasmids for ITS110.01 through ITS114.02 heavy and light chains generated by this study are available upon request for non-commercial research purposes from rosemarie.mason@nih.gov.

### Data and code availability

- The coordinates and maps for ITS110.01, ITS111.01, and IT114.01 have been deposited to the PDB with PDB: 9BP1, PDB: 9BLX, and PDB: 9BNS, respectively. The nucleotide sequences of heavy and light chains for these antibodies have been deposited in GenBank with codes GenBank: OR756535–OR756547 and GenBank: OR756548–OR756560.
- This paper does not report original code.
- Any additional information required to reanalyze the data reported in this paper is available from the lead contact upon request.

## STAR★METHODS

### EXPERIMENTAL MODEL AND STUDY PARTICIPANT DETAILS

#### Indian origin rhesus macaque specimens

SIV-positive plasma and peripheral blood mononuclear cells (PBMC) were obtained from previously completed animal study protocols. For non-human primate studies, the animals were housed and cared for in accordance with local, state, federal, and institute policies in an American Association for Accreditation of Laboratory Animal Care-accredited facility at a contract facility (Bioqual Inc., MD). All animal experiments were reviewed and approved by the Animal Care and Use Committee of the Vaccine Research Center, NIAID, NIH. Animal 08D213, a three-year old female monkey, was in the control group of a study to evaluate mucosal prime and systemic boost regimen (protocol VRC-08–238.2). It was vaccinated with rAd5-Null at 4×10^12^ viral particles through ileum, boosted 2 weeks later with rChAd3-Null 4×10^12^ viral particles through intramuscular injections. The animal was infected at six weeks postboost with SIVsmE660 through the intrarectal route (IR). Animal DBME was from a previously completed animal study protocol evaluating the effect of TRIM5 genotype on mucosal acquisition of SIVsmE660.^32^

### METHOD DETAILS

#### Peptide binding assays

The SIV MPR.03 biotinylated peptide containing lysines at both ends for solubility (KKKNEQELLELDKWASLWNWFDITNWLWYI RKKK-biotin) and HIV-1 MPER Scr.02 peptide were purchased from CPC Scientific Inc (San Jose, CA). Peptides were >98% pure as tested by HPLC. The following reagent was obtained through the NIH HIV Reagent Program, Division of AIDS, NIAID, NIH: Peptide Array, Simian Immunodeficiency Virus (SIV)mac239 Env Region, ARP-6883, contributed by DAIDS/NIAID. Binding of plasma samples to peptides was measured by enzyme-linked immunosorbent assay (ELISA) as previously described.^6^ Briefly, plates were coated with 2 μg/mL of peptide(s) dissolved in PBS at 4 °C overnight. Plates were then washed 3 times with PBS containing 0.1% Tween 20 (PBS-T) and blocked with 200 μL protein-free blocking buffer (Thermo Fisher). Serial dilutions of plasma were added to captured SIVmac239 Env peptide(s) in 100 μL protein-free blocking buffer and incubated for 1 h at 37 °C. After washing plates 3 times with PBS-T, binding was detected by HRP-conjugated anti-monkey IgG (Southern Biotech) at a 1:5,000 dilution for 1 h. The signal was developed by addition of 3,3′,5,5′ -tetramethylbenzidine (TMB) substrate (SureBlue; KPL) for 10 min. Reactions were terminated with 1 N sulfuric acid, and the optical density (OD) was read at 450 nm.

#### Virus neutralization and competition assays

Plasmid DNA encoding SIV gp160 was used in combination with a luciferase reporter plasmid containing the essential HIV structural genes to produce SIV Env pseudo-typed viruses as described previously.^6^ Briefly, virus neutralization was measured using single round infection of TZM-bl target cells by SIV Env pseudo-typed viruses. Titers were calculated as either the 50% inhibitory concentration (IC50) of mAbs or reciprocal plasma dilution (ID50) of plasma that caused a 50% reduction of relative light units (RLU) compared to results for the virus-treated or untreated control wells.^33^ Maximum percent neutralization (%VMax) was defined as the maximum % neutralization observed over the range of mAb concentrations or plasma dilutions tested.^6^

Competition of plasma neutralization was assessed as previously described.^34^ Briefly, the virus neutralization assay was performed by adding a fixed concentration (25 μg/mL) of soluble peptides to serial dilutions of plasma for 30 min prior to the addition of SIV Env pseudo-typed virus. Following addition of pseudo-typed virus, the mixture was incubated for 30 min at 37°C and TZM-bl cells were added at 0.5 million cells/ml and incubated for 48 h, followed by cell lysis and measurement of luciferase activity.

#### Isolation of SIV MPER-specific B cells by fluorescence-activated cell sorting (FACS)

Cryopreserved PBMC from SIV-infected rhesus macaques were thawed and stained with LIVE/DEAD Fixable Violet Dead Cell Stain (Life Technologies) as previously described.^6^ Cells were washed and stained with an antibody cocktail consisting of CD3 (clone SP34–2, BD Biosciences), CD4 (clone OKT4, BioLegend), CD8 (clone RPA-T8, BioLegend), CD14 (clone M5E2, BioLegend), CD20 (clone 2H7, BioLegend), IgG (G18–145, BD Biosciences) and IgM (clone G20–127, BD Biosciences) at room temperature in the dark for 20 min. The cells were washed twice with PBS and subsequently stained with fluorescently labeled SIV MPER peptide coupled separately to either ExtrAvidin–R-Phycoerythrin (PE) (Sigma) or streptavidin-allophycocyanin (APC) ThermoFisher). B cells (Live+CD3^−^CD4^−^CD8^−^CD14^−^CD20+IgM-IgG+) double-positive for both SIV MPER peptide-conjugated probes were sorted as single cells using BD FACSAria III sorter into individual wells of a 96-well plate containing reverse-transcriptase reaction buffer and plates were immediately sealed and stored at −80°C. Flow cytometric data was analyzed using FlowJo v9.

#### RT-PCR, cloning and expression of immunoglobulin genes

Rhesus macaque immunoglobulin (Ig) heavy and light chain variable regions were amplified from sorted B cells by reverse transcription polymerase chain reaction (RT-PCR) as previously described.^6^ Briefly, first-strand complementary DNA (cDNA) from B cells was synthesized using Superscript III Reverse Transcriptase (Life Technologies) and random hexamers (Gene Link). Nested PCR amplification of heavy and light chain variable regions was performed using HotStarTaq Plus DNA polymerase (QIAGEN) and rhesus-specific primers (Tables S1–3) as previously described.^6,35^ Amplified PCR products were analyzed on 2% agarose gels (Embi-Tec) and positive reactions sequenced by Sanger sequencing (ACGT Inc.). Antibody variable regions were codon optimized for human cell expression, synthesized (GenScript) and cloned into rhesus IgG, IgKappa and IgLambda expression vectors.^7^

#### Immunoglobulin gene family analysis

Antibody sequences were annotated in SONAR,^36^ using IMGT,^37^ IgDiscover,^38^ and a published macaque Ig database.^39^ Gene nomenclature follows that used in the database, since copy-number variation in the macaque Ig locus has been insufficiently explored to confidently order genes as the standard naming convention would require.

#### Generation of SIV MPER Fab and F(ab′)2

ITS110, ITS111, and ITS114 heavy and light chains were codon optimized for mammalian expression and altered by inserting an HRV3C recognition site (GLEVLFQGP) after Lys 235 of the heavy chain. These were cloned into plasmid pVRC8400 and transiently co-transfected into Expi293 cells using ExpiFectamine 293 Transfection Kit according to the manufacturer protocol. IgG was purified after 5 days using Protein A agarose (Pierce), dialyzed into PBS. Following expression and purification of mAbs, the IgG was subjected to HRV3C protease overnight at room temperature. Products were re-run over Protein A columns and the Fab flow-through was further purified by gel filtration. For F(ab′)2 fragments, SIV MPER mAb IgG was cleaved using Immobilized Pepsin (ThermoFisher) according to manufacturer’s directions. Briefly, 2mg of IgG was added to tube containing Immobilized Pepsin (50% slurry) in digestion buffer and incubated for 4 h at 37C with rotation. Digested F(ab′)2 was separated from the Immobilized Pepsin by centrifugation at 1,000 X g for 5 min, decanted into fresh tube, washed with 1.5 mL of 10mM Tris-HCl, pH 7.5, and then dialyzed into 1X PBS using Amicon Ultra Centrifugal Filter, 50 kDa MWCO (Millipore).

#### Crystal structures

Samples of each Fab were incubated with 5-fold molar excess SIV_mac239_ MPER peptide, residues 660–683 with three Arg residues added to the C terminus to increase solubility, referred to as SIV_mac239_-MPER-3R (LQKLNSWDVFGNWFDLASWIKYIQRRR), to Fab and screened for crystallization using 572 conditions from Hampton, Wizard and Precipitant Synergy screens using a Cartesian Honeybee or mosquito crystallization robot with 0.1μL of reservoir solution and 0.1 μL of protein solution per condition. Crystals were obtained for ITS110, ITS111, and ITS114, no crystals were obtained for other MPER Fabs. Crystals were flash-frozen in liquid nitrogen mother liquor supplemented with cryoprotectant. Data were collected at 1.00Å using the SER-CAT beamline ID-22 of the Advanced Photon Source, Argonne National Laboratory (Lemont, IL).

Diffraction data were processed with the HKL2000 suite 6.^40^ Molecular replacement solutions were obtained with Phaser in Phenix.^41^ The structures of the peptide-bound complexes were then manually adjusted, and peptide was built out using COOT,^42^ and was refined with Phenix. Density beyond what was built for SIV_mac239_-MPER-3R peptide was visible but not suitable for accurate modeling, only regions with clear density were included in the model.

#### Generation of anti-idiotype antibody

The αITS113-Id1 anti-idiotype antibody was generated and tested as described previously.^9^ Briefly, mice were vaccinated with the ITS113.01 Fab and Fab-specific B cells were single-cell sorted. Antibody immunoglobulin genes were amplified by nested PCR and sequenced by Sanger sequencing (ACGT Inc.). Sequenced antibody variable regions were synthesized (GenScript) and cloned into murine heavy and light chain antibody expression vectors. Antibodies were expressed in Expi293F cells via transient transfection with ExpiFectamine 293 (Thermo Fisher). Antibodies were purified from the supernatant using Protein G Sepharose 4 Fast Flow (Cytiva). Binding of the αITS113-Id1 antibody to rhesus and human mAbs was measured by ELISA. ELISA plates were coated with either the αITS113-Id1 antibody or purified mAbs. Following blocking, purified rhesus or human mAbs (for αITS113-Id1 coating) or αITS113-Id1 (for mAb coating), were titrated. Binding was detected using either an HRP anti-mouse secondary (Jackson Immunoresearch; cat no 209-035-098, 1:5000 dilution) or an anti-rhesus IgG Fc-HRP (Southern Biotech; clone: SB108a).

### QUANTIFICATION AND STATISTICAL ANALYSIS

General quantitation and representation throughout the manuscript was performed using GraphPad Prism v8 and Microsoft Excel. The number of NHP initially screened for MPER peptide binding was 14. Neutralization assays were performed in duplicate or triplicate and the experimental details can be found in the STAR Methods. Crystallographic quantitation in Table 1 was achieved through Phenix.

## Supplementary Material

1

## Figures and Tables

**Table 1.** Crystallographic data collection and refinement statistics

	ITS110.01 with SIV MPER peptide	ITS111.01 with SIV MPER peptide	ITS114.01 with SIV MPER peptide
PDB ID	9BP1	9BLX	9BNS
Data collection			
Space group	P62	C2	P6522
Cell dimensions			
a, b, c (Å)	210.2, 210.2, 46.2	166.9, 118.8, 66.5	175.9, 175.9, 206.0
a, b, g (°)	90, 90, 120	90, 104.7, 90	90, 90, 120
Resolution (Å)	40.0–3.6 (3.66–3.6)	40.0–2.0 (2.03–1.96)	40–3.0 (3.07–3.00)
*R*_*merge*_	0.17 (1.26)	0.23 (0.59)	0.14 (1.97)
*I/sI*	17.5 (1.4)	6.3 (1.8)	7.33 (0.66)
Completeness (%)	95.6 (88.7)	96.8 (80.9)	95.44 (96.82)
Redundancy	2.9 (2.0)	3.9 (2.2)	4.8 (4.9)
Refinement			
Resolution (Å)	39.72–3.58 (3.71–3.58)	33.94–1.96 (2.01–1.96)	45.54–3.0 (3.12–3.00)
No. reflections	12,088 (410)	76,064 (3442)	36,533 (3,626)
*R*_*work*_*/R*_*free*_ (%)	20.6/26.55	17.6/20.9	26.8/30.7
No. atoms (total)	6,792	7,490	5,529
Protein	6,792	6,862	5,529
Water	0	577	0
Ligand	0	120	0
B factors (Å)^2^			
Protein	87.52	37.04	128.2
Water	N/A	43.08	N/A
RMSDs			
Bond lengths (Å)	0.004	0.007	0.006
Bond angles (°)	0.57	0.97	1.2

Values in parentheses are for the highest-resolution shell. One crystal was used to determine each structure.

**Table:** KEY RESOURCES TABLE

REAGENT or RESOURCE	SOURCE	IDENTIFIER
Antibodies		
Monoclonal anti-human CD3-BV421, clone SP34-2	BD Biosciences	Cat#562877; RRID:AB_2737860
Monoclonal anti-human CD4-BV421, clone OKT4	Biolegend	Cat#317433; RRID:AB_11150413
Monoclonal anti-human CD8-BV421, clone RPA-T8	Biolegend	Cat#301036; RRID:AB_10960142
Monoclonal anti-human CD14-BV421, clone M5E2	Biolegend	Cat#301830; RRID:AB_10959324
Monoclonal anti-human CD20-PerCP-Cy5.5, clone 2H7	Biolegend	Cat#302326; RRID:AB_893283
Monoclonal anti-human IgG-AlexaFluor 680, clone G18-145	Mario Roederer, NIH	N/A
Monoclonal anti-human IgM-FITC, clone G20-127	BD Biosciences	Cat#555782; RRID:AB_396117
Bacterial and virus strains		
SIV virus panel	NIH/VRC	N/A
Biological samples		
PBMC from animals 08D213 and DBME	NIH/VRC	N/A
Serum from animals 08D213 and DBME	NIH/VRC	N/A
Chemicals, peptides, and recombinant proteins		
LIVE/DEAD^®^ Fixable Violet Dead Cell Stain	Thermo Fisher	Cat#L34955
CompBead Anti-Mouse Ig, Compensation Particles	BD Biosciences	Cat#552843
Peptide Array, SIVmac239 Env Region	NIH HIV Reagent Program, Division of AIDS, NIAID, NIH	ARP-6883
MPER.03.SIVmac239 peptide	CPC Scientific	N/A
HIV-1 MPER.Scr.02 peptide	CPC Scientific	N/A
Streptavidin, R-phycoerythrin (SA-PE)	Thermo Fisher	Cat#S866
Streptavidin-allophycocyanin (SA-APC)	Thermo Fisher	Cat#S868
RNAse OUT	Thermo Fisher	Cat#10777019
Random Hexamers	Gene Link	Cat#26-4000-03
10mM dNTP mix	Bioline	Cat#BIO-39053
Carrier RNA	Qiagen	Cat#1017647
Mouse Anti-Monkey IgG-HRP	Southern Biotech	Cat #4700-05
SureBlue TMB Peroxidase Substrate	KPL	Cat#52-00-03
1 N sulfuric acid	Fisher Scientific	Cat#SA212-1
DEAE-Dextran	Sigma	Cat#D9885-10G
Protein A Plus Agarose	Pierce	Cat#22811
FuGene 6 transfection reagent	Promega	Cat#E2961
Opti-MEM^™^ reduced serum media	Thermo Fisher	Cat#31985062
Immobilized Pepsin (Agarose Resin)	Thermo Fisher	Cat#20343
Critical commercial assays		
ExpiFectamine^™^ 293 Transfection Kit	ThermoFisher Scientific Inc.	Cat# A14525
BirA Biotin-Protein Ligase Bulk Reaction Kit	Avidity	Cat#bulk BirA
Superscript III Reverse Transcriptase Kit	Thermo Fisher	Cat#18080093
HotStarTaq Plus DNA Polymerase Kit	Qiagen	Cat#203603
Steadylite Plus reporter gene assay system	Perkin Elmer	Cat#6066759
Deposited data		
ITS110.01-peptide structure	This paper	PDB: 9BP1
ITS111.01-peptide structure	This paper	PDB: 9BLX
ITS114.01-peptide structure	This paper	PDB: 9BNS
ITS110.01 through ITS114.02 heavy chain nt sequences	This paper	GenBank: OR756535-OR756547
ITS110.01 through ITS114.02 light chain nt sequences	This paper	GenBank: OR756548-OR756560
Experimental models: Cell lines		
Human: HeLa-derived TZM-bl	NIH HIV Reagent Program	Cat# 8129-442, RRID: CVCL_B478
Human: Expi293F	ThermoFisher	Cat# A14527
Human: HEK 293T	ATCC	Cat#CRL-3216, RRID: CVCL_0063
Oligonucleotides		
Rhesus immunoglobulin primers	This publication	Table S1
Recombinant DNA		
pVRC8400 vector	https://www.addgene.org	Cat# 63160
CMVR rhesus IgG expression plasmid	NIH/VRC	N/A
CMVR rhesus IgK expression plasmid	NIH/VRC	N/A
CMVR rhesus IgL expression plasmid	NIH/VRC	N/A
pSG3DEnv backbone plasmid	NIH/VRC	N/A
SIVmac251.H9.15	David Montefiori	N/A
SIVmac251.30	David Montefiori	N/A
SIVmac239	David Montefiori	N/A
SIVsmE660.CP3C.A8	David Montefiori	N/A
SIVsmE660.CR54.2A5	David Montefiori	N/A
SIVsmE660.11	David Montefiori	N/A
SIVmac239.cs.23	David Montefiori	N/A
SIVmac251.6	David Montefiori	N/A
SIVmac251.cs.41	David Montefiori	N/A
SIVsm FFv 18Nov04 ENVPL2.1	Cynthia Derdeyn	N/A
SIVsm FJv 15Nov06 ENVPL2.1	Cynthia Derdeyn	N/A
SIVsm FWk 12Aug04 ENVPL4.1	Cynthia Derdeyn	N/A
SIVsm RSo8 17Jan06 ENVPL1.1	Cynthia Derdeyn	N/A
SIVmac251 RZj5 9Apr09 ENVPL2.1	Cynthia Derdeyn	N/A
SIVsmm RM174.V1.tf	George Shaw	N/A
SIVsmm RM174.V2.tf	George Shaw	N/A
SIVsmm RM174.V3.tf	George Shaw	N/A
SIV Outlier SL92b	George Shaw	N/A
Software and algorithms		
GraphPad Prism Software	GraphPad Prism Software, Inc.	N/A
Phenix	Phenix	phenix-online.org
FlowJo v.9.9.6	FlowJo, LLC	www.flowjo.com
IMGT/V-QUEST	International ImMunoGeneTics Information System; Marie-Paule Lefranc (Marie-Paule.Lefranc@igh.cnrs.fr), University of Montpellier, France	www.imgt.org

## Highlights

- Isolated four lineages of SIV MPER nAbs from two NHPs through targeted sera screening
- SIV MPER nAbs are broadly neutralizing but low in potency
- Structures and genetics indicate four lineages comprise a multi-donor antibody class
- SIV MPER nAbs bind the MPER shared by nAbs elicited in a human clinical trial
