# Supplementary material for: Isolation and structure of broad SIV-neutralizing antibodies reveal a proximal helical MPER epitope recognized by a rhesus multi-donor class
Source: Cell Rep. Author manuscript; Available in PMC 2025 Apr 9. (PMC11979902; doi:10.1016/j.celrep.2024.115163)
Supplement: 1 [file NIHMS2052780-supplement-1.pdf]

**Cell Reports, Volume *44***

## Supplemental information

# Isolation and structure of broad SIV-neutralizing antibodies reveal a proximal helical MPER epitope recognized by a rhesus multi-donor class

**Jason Gorman, Renguang Du, Yen-Ting Lai, Mohammed S. Ahmadi, Hannah A.D. King, Kaimei Song, Kimberly Manalang, Christopher A. Gonelli, Chaim A. Schramm, Cheng Cheng, Richard Nguyen, David Ambrozak, Aliaksandr Druz, Chen-Hsiang Shen, Yongping Yang, Daniel C. Douek, Peter D. Kwong, Mario Roederer, and Rosemarie D. Mason**

Cell Reports, Volume ■■

## Supplemental information

# Isolation and structure of broad SIV-neutralizing antibodies reveal a proximal helical MPER epitope recognized by a rhesus multi-donor class

**Jason Gorman, Renguang Du, Yen-Ting Lai, Mohammed S. Ahmadi, Hannah A.D. King, Kaimei Song, Kimberly Manalang, Christopher A. Gonelli, Chaim A. Schramm, Cheng Cheng, Richard Nguyen, David Ambrozak, Aliaksandr Druz, Chen-Hsiang Shen, Yongping Yang, Daniel C. Douek, Peter D. Kwong, Mario Roederer, and Rosemarie D. Mason**

**A**

```
IGHD6-33*01    ----SSGW-------------------
IGHJ6-6*01     -----------YGLDSWGQGVVVTVSS
ITS110.01H     CARHSSGWFSLYGLDSWGQGVVVTVSS
ITS110.02H     CARHSSGWFSLYGLDSWGQGVVVTVSS
ITS110.03H     CARHSSGWFSLYGLDLWGQGVVVTVSS
ITS110.04H     CARHSSGWFSLYGLDSWGQGVVVTVSS

IGHD3-18*01    -----SGYYT-----------------
IGHJ6-6*01     -----------YGLDSWGQGVVVTVSS
ITS111.01H     CARHSSGYFTLYALDSWGQGVVVTVSS
ITS111.02H     CARHSSGYFTLYGLDSWGQGVVVTVSS

IGHD2-12*01    -----SGI-------------------
IGHJ6-6*01     -----------YGLDSWGQGVVVTVSS
ITS112.01H     CARHSSGFFSLYALDPWGQGVGVSVSS
ITS112.02H     CARHSSGFFSLYALDSWGQGVVVSVSS
ITS112.03H     CAKHSSGFFSLYALDSWGQGVVVSVSS
```

**B**

```
IGHV4-AFQ-U*01 TGTGCGAGACA----------------------------------------
IGHD5-24*01    -----------------GGAGATACAGTGGGTACACTTAC-----------
IGHJ5-1*01     -------------------------------ACAACCGGTTCGATGTCTGG
ITS113.01H     TGTACGAGACAGGACCAGACACTACTCTGGGTGAGCCGGTTCACTGTCTGG
ITS113.02H     TGTACGAGACAGGACCAGACACTACTGTGGGTGAGCCGGTTCACTGTCTGG
ITS114.01H     TGTACGAGACAGGAGCAGACTCTATTTTGGGTCAAGCGGTTCGATGTCTGG
ITS114.02H     TGTACGAGACAGGAACAGACATTATTTTGGGTCAAGCGGTTCGATGTCTGG

IGHV4-AFQ-U*01 TGTGCGAGACA----------------------------------------
IGHD4-23*01    ---------TGAATACAGTAACTAC--------------------------
IGHJ5-1*01     -------------------------------ACAACCGGTTCGATGTCTGG
ITS113.01H     TGTACGAGACAGGACCAGACACTACTCTGGGTGAGCCGGTTCACTGTCTGG
ITS113.02H     TGTACGAGACAGGACCAGACACTACTGTGGGTGAGCCGGTTCACTGTCTGG
ITS114.01H     TGTACGAGACAGGAGCAGACTCTATTTTGGGTCAAGCGGTTCGATGTCTGG
ITS114.02H     TGTACGAGACAGGAACAGACATTATTTTGGGTCAAGCGGTTCGATGTCTGG

IGHV4-AFQ-U*01 TGTGCGAGACA----------------------------------------
IGHD2-33*01    -------------AGCACACTGTAGTGATAGTGGCTGCTCCTCC-------
IGHJ5-1*01     -------------------------------ACAACCGGTTCGATGTCTGG
ITS113.01H     TGTACGAGACAGGACCAGACACTACTCTGGGTGAGCCGGTTCACTGTCTGG
ITS113.02H     TGTACGAGACAGGACCAGACACTACTGTGGGTGAGCCGGTTCACTGTCTGG
ITS114.01H     TGTACGAGACAGGAGCAGACTCTATTTTGGGTCAAGCGGTTCGATGTCTGG
ITS114.02H     TGTACGAGACAGGAACAGACATTATTTTGGGTCAAGCGGTTCGATGTCTGG

IGHV4-AFQ-U*01 TGTGCGAGACA----------------------------------------
IGHD3-34*01    ---------GTACTGGGGTGATTATTATGAC--------------------
IGHJ5-1*01     -------------------------------ACAACCGGTTCGATGTCTGG
ITS113.01H     TGTACGAGACAGGACCAGACACTACTCTGGGTGAGCCGGTTCACTGTCTGG
ITS113.02H     TGTACGAGACAGGACCAGACACTACTGTGGGTGAGCCGGTTCACTGTCTGG
ITS114.01H     TGTACGAGACAGGAGCAGACTCTATTTTGGGTCAAGCGGTTCGATGTCTGG
ITS114.02H     TGTACGAGACAGGAACAGACATTATTTTGGGTCAAGCGGTTCGATGTCTGG
```

**C**

```
IGKV1-AAY*01   TGTCAACAGTATAACAGTGACCCTCC-------
IGKJ3*01       -----------------------ATTCACTTTC
ITS113.01K     TGTCAACACTATTACAGTGACCCATTCACTTTC
ITS113.02K     TGTCAGCAGTATTACAGTGACCCATTCACTTTC
ITS114.01K     TGTCAACAATATTACAGTGACCCATTCACTTTC
ITS114.02K     TGTCAACAGTATTACAGTGACCCATTCACTTTC
```

**Figure S1. Junctional analysis of SIV MPER antibodies, related to Figure 2.**

(A) Protein translation of best DNA matched D- and J-gene assignments from KIMDB for the ITS110, ITS111, ITS112 lineages. A number of D genes showed close probability but assignments for all clones were consistent within lineages.

(B) Possible D-gene assignments for the ITS113/114 lineage. Red strikeout indicates bases putatively subjected to germline excision. Grey indicates unmutated germline-encoded bases; blue indicates germline-encoded bases that have been mutated. The underlined segment is putatively derived from the D gene in each scenario. Green indicates non-templated bases that are conserved in all 4 antibodies; purple indicates non-templated nucleotides with variation. Although the optimal assignment for each antibody in isolation varies, overall conservation suggests they are more likely than not to be derived from the same rearrangement event.

(B) Light chain junctions are highly similar but could be coincidental due to low diversity. Colors are the same as in (B).

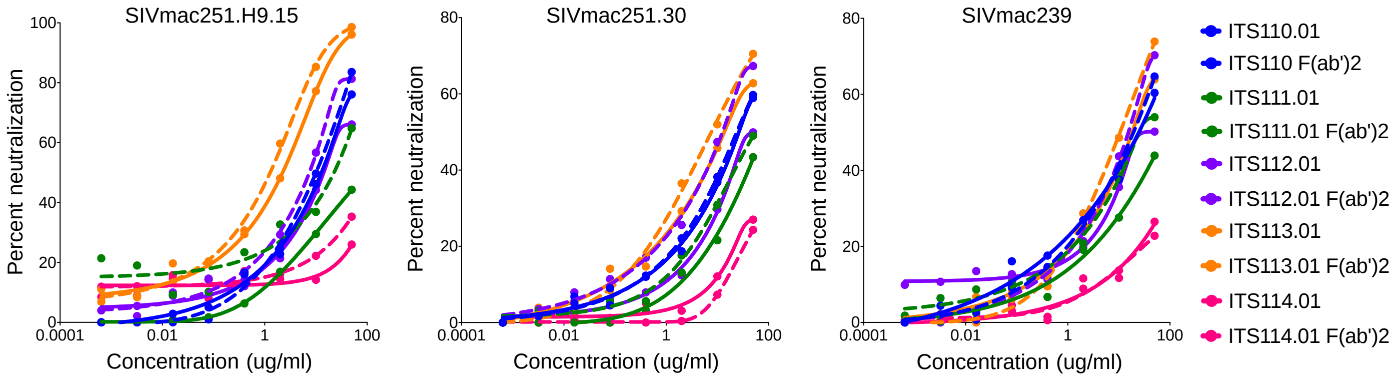


**Figure S2. F(ab′)2 fragment neutralization, related to Figure 2.**
F(ab′)2 fragments of mAbs from each lineage were assessed for virus neutralization compared to their IgG equivalent. There was no significant increase in neutralization potency of SIV by F(ab’)2 fragments (dashed line) compared to IgG (solid line).

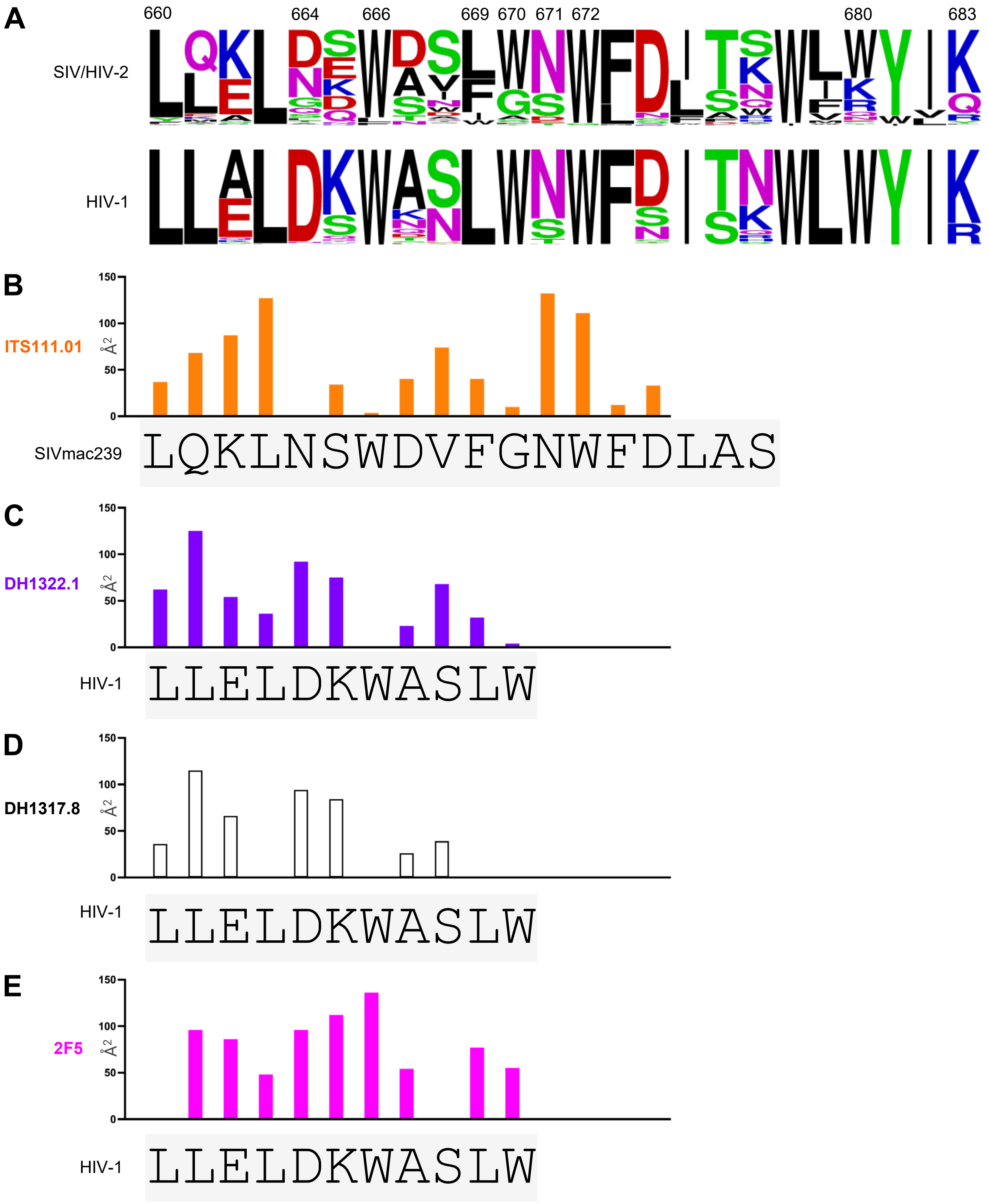


**Figure S3. MPER epitope buried surface area, related to Figures 4 and 5.**

(A) The Logo plots for SIV/HIV-2 and HIV-1 are shown as in Figure 5C for reference to aligned exemplar sequences for the structures below.

(B) The buried surface area for ITS111.01 is shown as a bar graph above the co-crystalized peptide sequence.

(C) The buried surface area for 2F5 (PDB ID 3MOA) is shown as a bar graph above the co-crystalized peptide sequence.

(D) The buried surface area for DH1322.1 (PDB ID 8G8C) is shown as a bar graph above the co-crystalized peptide sequence. Only the overlapping epitope is shown, omitting several residues at the N –terminal of the peptide.

(E) The buried surface area for DH1317 (PDB ID 8G8A) is shown as a bar graph above the co-crystalized peptide sequence. Only the overlapping epitope is shown, omitting several residues at the N –terminal of the peptide.

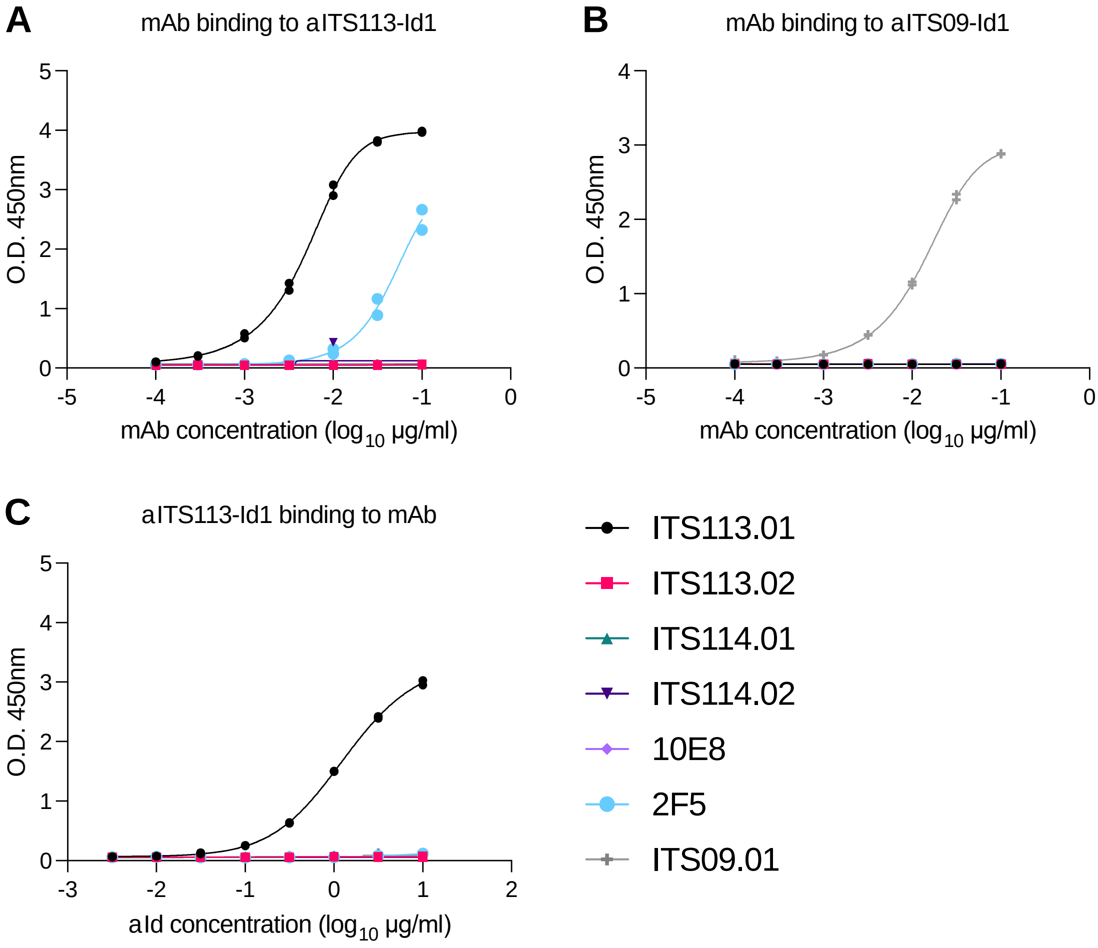


**D**

**αITS113-Id1 Heavy**

EVQLQESGAELVRPGASVKMSCKASGYTFTSYNMHWVKQTPRQGLEWIGAIYPGNGDTSYNQKFRGKATLIVDKSSSTV
YMQLSSLTSEDSAVYFCARGDGDYFDYWGQGTTLTVSS

**αITS113-Id1 Light**

DIVMTQSPSSLAVSVGEKVTMSCKSSQSLLYSSNQQNYLAWYQQKPGQSPKLLIYWASTRESGVPDRFTGSGSGTDFTL
TISSVKAEDLAVYYCQQYYSYPPTFG

**Figure S4. Anti-ITS113 idiotype antibody binding specifically to ITS113.01, related to Figure 5.**

(A) Binding of purified αITS113-Id1 to mAbs by ELISA. ELISA plates were coated with 1 µg/ml αITS113-Id1 and rhesus/human mAbs were titrated at the concentrations indicated.

(B) Binding as in panel (A) but plates were coated with αITS09-Id1.

(C) ELISA plates were coated with 1 µg/ml rhesus or human mAbs, and αITS113-Id1 was titrated at the concentrations indicated. The color key applies to A-C.

(D) Sequence of αITS113-Id1 to mAb Heavy and Light variable region.

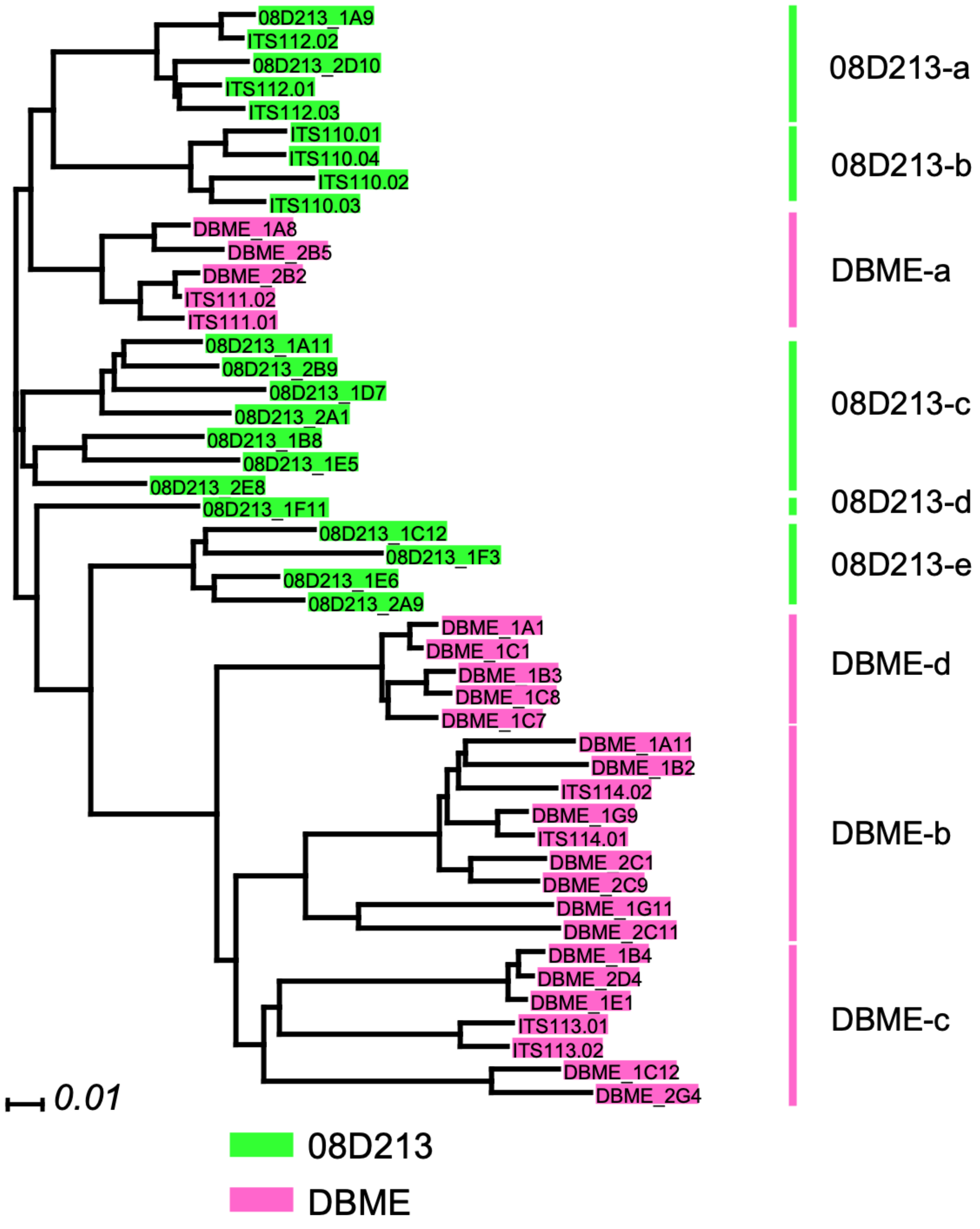


**Figure S5. Phylogenetic tree of concatenated heavy and light chain protein sequences.**
Phylogenetic tree of concatenated heavy and light chain sequences from neutralizing and non-neutralizing mAbs isolated from animals 08D213 and DBME. Neutralizing mAbs are indicated using ITS nomenclature while non-neutralizing mAbs are indicated by animal ID and sort well number. The bar at the bottom of the phylogenetic tree represents the length of branch representing a genetic change of 0.01. Summary of sequences used for analysis are listed in Table S4.

**Table S1. Rhesus Ig heavy chain primers, related to Figure 2.**

| RhH-O (1st round (O)uter primers) | |
|---|---|
| Primer name | Sequence (5'-3') |
| Forward primers | |
| RhH1-O | ATGGACTKGACCTGGAGG |
| RhH2-O | ATGGACTTGACCTGGAAG |
| RhH3-O | ATGGACTGGACCTGGAG |
| RhH4-O | ATGGACACGCTTTGCTCC |
| RhH5-O | ATGGAGTTTGGGCTGAGC |
| RhH6-O | ATGGAGTTTGGACTGAGC |
| RhH7-O | ATGGAGTTGGGACTGAGC |
| RhH8-O | ATGGAGTCGTGGCTGAG |
| RhH9-O | ATGGAGTTGGGGCTGAG |
| RhH10-O | GGAATTTAGGCTGAGCTG |
| RhH11-O | ATGGAATTTGGGCTGAGC |
| RhH12-O | GAAACACCTGTGGTTCTT |
| RhH13-O | ATGAAGCACCTGTGGGTC |
| RhH14-O | ATGAAGCACCTGTGGTTC |
| RhH15-O | ATGGGGTCAACTGCCATC |
| RhH16-O | ATGGAGTTKGGGCTGAGC |
| RhH17-O | ATGGAGTTTGKRCTGAGC |
| RhH18-O | ATGGAGTCRTGGCTGAGC |
| RhH19-O | ATGGAGTTTGTGCTGAGT |
| RhH20-O | ATGGGGTCCACCGTCAC |
| RhH21-O | ATGTCTGTCTCCTTCCTC |
| RhH22-O | ACTGGGCTGGGAGGATC |
| RhH23-O | ATGGACTGGATATGGAGGATC |
| RhH24-O | ATGGACACGCTTTGCTAC |
| RhH25-O | AGTAGCACATACTACCCAGAC |
| RhH26-O | CACTCTGTGGTTTGAGATAGACG |
| RhH27-O | GCTGAGCTGGGTTTTCCTTC |
| RhH28-O | ATGGAATTTTGGCTGAGCTG |
| RhH29-O | ATGGAGTTCGGGCCGAG |
| RhH30-O | ATGGAGTCTGGGCTGAGC |
| RhH31-O | ATGAAGTTTGGGCTGAGC |
| RhH32-O | ATGGAGTTTGGCCTGAGC |
| RhH33-O | CCATGGAGTTTATGCTGAGC |
| RhH34-O | ATGGACCTCACCTGGAGC |
| Reverse primers | |
| 3CgCH1 | GGAAGGTGTGCACGCCGCTGGTC |
| 3RhCgCH1 | AGGTGTGCACGCCGCTGG |

| RhH-O (2nd round (I)nner primers) | |
|---|---|
| Primer name | Sequence (5'-3') |
| Forward primers | |
| RhH1-I | GCCCAGTCCCAGGTCCAG |
| RhH2-I | GTCCTGTCACAGGTGCAGCTG |
| RhH3-I | GCCCACTCCCAGGTGCAG |
| RhH4-I | GTCCAGTCCCAGGTCCAGC |
| RhH5-I | GCCCACTCCCTGGTGCAG |
| RhH6-I | GCCCACTCCGAGGTCCAG |
| RhH7-I | CGCCCACTCTGAGGTCCAG |
| RhH8-I | GGTGTCCAGTCCCAAGTCCAAC |
| RhH9-I | GGTCCTGTCCCAGGTGCAG |
| RhH10-I | GGGTCCTGTCCCAGGTGAAG |
| RhH11-I | TGTCCTGTCACAGGTGCAGC |
| RhH12-I | GTCCTGTCCCTGGTGCAGC |
| RhH13-I | GTCCTGTCCCTGGTGCACC |
| RhH14-I | GTCCTGTCCCAGCTGCAGC |
| RhH15-I | GGGTCTTGTCCCAGGTGCAC |
| RhH16-I | GGTGTCCTGTCACAGGTGCAG |
| RhH17-I | GGGTCCTGTCACAGGTGCAG |
| RhH18-I | GGGTCTTGTCCCAGGTGCAG |
| RhH19-I | GGGTAGTGTCCCAGGTGCAA |
| RhH20-I | GGCCTGTCCCAGGTGCAG |
| RhH21-I | GGGTCTTGTCCCAGGTGACC |
| RhH22-I | GGCTCTTGTCCCAGGTGACC |
| RhH23-I | GGGTCTTCTCCCAGGTGACC |
| RhH24-I | GGGTCCTGTCCCAGGTACAG |
| RhH25-I | GGGTCCTGTCCCAGGTACAAC |
| RhH26-I | GGTCCTGTCCCAGGTGCAC |
| RhH27-I | GGTCCTGTCCCAGGTGCAA |
| RhH28-I | GAGTCTGTGCCGAGGTGCAG |
| RhH29-I | GGTGTCCAGTGTGACGTGCAG |
| RhH30-I | GGTGTCCAGTGTGAAGTGCAG |
| RhH31-I | GGTGTCCAGTGTGAGGTGCAG |
| RhH32-I | GGTGTCCAGTGTGAGGTGCAA |
| RhH33-I | GGTGTCCAGTGTGAGATGCAGC |
| RhH34-I | GGTGTCCAGTGTGAGGTGCA |
| RhH35-I | GGTGTCCAGTGTGAGGTGCG |
| RhH36-I | GGTGTCCAGTGTGAGGTGAAGTTG |
| RhH37-I | GGGTCTTGTCCCAGATGCAGC |
| RhH38-I | GGTCCTGTCCCAGCTGCAG |
| RhH39-I | TCCCAGTGTGAGGTGCAGC |
| RhH40-I | GGGGTTTTGTCCCAGGTGACC |
| RhH41-I | GGGGTTCAGTGTGAGGTGCAG |
| RhH42-I | GGTGTGCAGTGTGAGGTGCAG |
| RhH43-I | GGTGCCCAGTGTGAGGTGCAG |
| RhH44-I | GGTGTCCAGTGTGTGGAGCAG |
| RhH45-I | GTGTCTCTGCCGAGGTGCAG |
| RhH46-I | GCTACTTTAAGAGGTGTCCTGTGTGAGG |
| RhH47-I | CTAAAAGGTGTCCAGTGTGCGGTAG |
| RhH48-I | TTAAAAGGTGTCCAGGGTGAGGTCC |
| RhH49-I | CAGCTACAGGTGCCCAGTCTC |
| RhH50-I | AAAAGCAGCCCAGCCCTG |
| RhH51-I | GTGTCCGGTGTGAGGTGCAG |
| RhH52-I | GGTGTCAAGTGTGAGGCGCAG |
| RhH53-I | AGTTGTCCAGTGTGAGGTGCAG |
| RhH54-I | GAAGGTGTGAGTTCTGAGGTGAGATTGG |
| Reverse primer | |
| 3IgGInt | GTTCGGGGAAGTAGTCCTTGAC |

**Table S2. Rhesus Ig kappa chain primers, related to Figure 2.**

| **RhLk-O (1st round (O)uter primers)** | |
|---|---|
| Primer name | Sequence (5'-3') |
| Forward primers | |
| RhLk1-O | ACCACCGGAGAAATAGTGATG |
| RhLk2-O | ACCACCGGAGAAATCGTG |
| RhLk3-O | CCAGGTGCCAGATGCG |
| RhLk4-O | CCAGGTGCCCGATGTG |
| RhLk5-O | CCAGGTGCCAGATGTGAC |
| RhLk6-O | ACCACCGGAGAAATTGTGTTG |
| RhLk7-O | CAGGTGCCCAGATGTGAC |
| RhLk8-O | GCCTGCGGGGACATTG |
| RhLk9-O | GCCTACGGGGACATTGTG |
| RhLk10-O | GCCTGTGGGGACATTGTG |
| RhLk11-O | GCCTACGGGGACATCG |
| RhLk12-O | ATGGGGACATTGTGTTGACC |
| RhLk13-O | TCCAGTGGGGATATTGTGATGAC |
| RhLk14-O | GCCTCCAAGGGTGAAATTGTG |
| RhLk15-O | TCCAGTGGGGATGTTGTGATAAC |
| RhLk16-O | TCCAGTGGGGATGTTGTG |
| RhLk17-O | ACCACCGGAGAAATAGTGTTG |
| RhLk18-O | CGGAGGAGAAATTGTGATGCAG |
| RhLk19-O | ACCGGAGAAACAGTGGTG |
| RhLk20-O | ACCACCGGAGAAATTGTATTGAC |
| RhLk21-O | CGGCGGAGAAATTGTGATGC |
| RhLk22-O | TCAGGTGCCAGATGTGAC |
| RhLk23-O | TCAGGTGCCAAATGTGACATC |
| RhLk24-O | TCAGGTGCAAGATGTGACATC |
| RhLk25-O | TCCAGTGGGGATATTGTGATGATC |
| RhLk26-O | CGGTGGGGATATTGTGATGAC |
| RhLk27-O | TCCAGTGCGGATATTGTGATG |
| RhLk28-O | TCCAGTGGGGATGTTGTAATG |
| RhLk29-O | AGTGGGGATGTTGTGATGAC |
| RhLk30-O | ACTGGGGATGTTGCGATG |
| RhLk31-O | AGTGGGGATACTGTGATGACC |
| RhLk32-O | ACCACTGGAGAAATTGTAATGACG |
| RhLk33-O | ACCACTGGAGAAATTGTGATGAC |
| RhLk34-O | CGCCGGAGAAATTGTAATGAC |
| RhLk35-O | ACCACCGGACAAGTTATATTGAC |
| Reverse primer | |
| RhLkrev-O | TACGCTGGCCTCTCTGGGATAGAAG |

| **RhLk-I (2nd round (I)uter primers)** | |
|---|---|
| Primer name | Sequence (5'-3') |
| Forward primers | |
| RhLk1-I | ACCACCGGAGAAATAGTGATGACGCAG |
| RhLk2-I | ACCACCGGAGAAATCGTGATGACG |
| RhLk3-I | CCAGGTGCCAGATGCGACATTCAG |
| RhLk4-I | CCAGGTGCCCGATGTGACATTCAG |
| RhLk5-I | CCAGGTGCCAGATGTGACATTCAG |
| RhLk6-I | CCAGGTGCCAGATGTGACATCCAG |
| RhLk7-I | ACCACCGGAGAAATTGTGTTGACG |
| RhLk8-I | CAGGTGCCCAGATGTGACATTCAG |
| RhLk9-I | GCCTGCGGGGACATTGTGATGAC |
| RhLk10-I | GCCTACGGGGACATTGTGATGACC |
| RhLk11-I | GCCTGTGGGGACATTGTGATGACC |
| RhLk12-I | GCCTACGGGGACATCGTGATGAC |
| RhLk13-I | ATGGGGACATTGTGTTGACCCAGTC |
| RhLk14-I | TCCAGTGGGGATATTGTGATGACCCAG |
| RhLk15-I | CCAGGTGCCAGATGTGACATTCAGATG |
| RhLk16-I | CCAGGTGCCAGATGTGACATACAG |
| RhLk17-I | GCCTCCAAGGGTGAAATTGTGATGACTC |
| RhLk18-I | TCCAGTGGGGATGTTGTGATAACTCAG |
| RhLk19-I | TCCAGTGGGGATGTTGTGATGAATCAG |
| RhLk20-I | TCCAGTGGGGATGTTGTGATGACTCAG |
| RhLk21-I | ACCACCGGAGAAATAGTGTTGATGCAG |
| RhLk22-I | CGGAGGAGAAATTGTGATGCAGCAG |
| RhLk23-I | ACCGGAGAAACAGTGGTGACGC |
| RhLk24-I | ACCACCGGAGAAATTGTATTGACGCAG |
| RhLk25-I | CGGCGGAGAAATTGTGATGCAGC |
| RhLk26-I | TCAGGTGCCAGATGTGACATTCAG |
| RhLk27-I | TCAGGTGCCAAATGTGACATCCAG |
| RhLk28-I | TCAGGTGCAAGATGTGACATCCAG |
| RhLk29-I | TCCAGTGGGGATATTGTGATGATCCAG |
| RhLk30-I | CGGTGGGGATATTGTGATGACCCAG |
| RhLk31-I | TCCAGTGCGGATATTGTGATGACCCAG |
| RhLk32-I | TCCAGTGGGGATGTTGTAATGACTCAG |
| RhLk33-I | AGTGGGGATGTTGTGATGACCCAG |
| RhLk34-I | ACTGGGGATGTTGCGATGACTCAG |
| RhLk35-I | AGTGGGGATACTGTGATGACCCAG |
| RhLk36-I | ACCACTGGAGAAATTGTAATGACGCAG |
| RhLk37-I | ACCACTGGAGAAATTGTGATGACGCAG |
| RhLk38-I | CGCCGGAGAAATTGTAATGACGCAG |
| RhLk39-I | ACCACCGGACAAGTTATATTGACTCAG |
| Reverse primer | |
| RhLkrev-I | CAGCAGGCACACAACAGAGACAG |

**Table S3. Rhesus Ig lambda chain primers, related to Figure 2**.

| **RhLl-O (1st round (O)uter primers)** | | **RhLl-I (2nd round (I)nner primers)** | |
|---|---|---|---|
| Primer name | Sequence (5'-3') | Primer name | Sequence (5'-3') |
| Forward primers | | Forward primers | |
| RhLl1-O | TCCTGGGCCCAGTCTG | RhLl1-I | TCCTGGGCCCAGTCTGCG |
| RhLl2-O | TCCTGGGCCCAGTTTG | RhLl2-I | TCCTGGGCCCAGTTTGTGC |
| RhLl3-O | TCCTTGGCCCAGTCTGTC | RhLl3-I | TCCTTGGCCCAGTCTGTCCT |
| RhLl4-O | TCCTGGGCGCAGTCTG | RhLl4-I | TCCTGGGCCCAGTCTGTCC |
| RhLl5-O | TCCTGGTCCCAGTCTGTG | RhLl5-I | TCCTGGGCGCAGTCTGTGC |
| RhLl6-O | TCCTGGGGCCAGTCTG | RhLl6-I | TCCTGGGCCCAGTCTGCC |
| RhLl7-O | TCCTGGGCCCAGCCTG | RhLl7-I | TCCTGGGCCCAGTCTGTC |
| RhLl8-O | TCCCTCTCCCAGCCTG | RhLl8-I | TCCTGGGCCCAGTCTGTGC |
| RhLl9-O | TCTCTCTCCCAGCCTGTG | RhLl9-I | TCCTGGTCCCAGTCTGTGCTG |
| RhLl10-O | CAGGTTCTGTGGTTTCTTCTGAG | RhLl10-I | TCCTGGGGCCAGTCTGCC |
| RhLl11-O | GGTTCCGTGGTTTCCTCTG | RhLl11-I | TCCTGGGCCCAGTTTGTGCT |
| RhLl12-O | CAGTCTCTGTGGCCTCCTATG | RhLl12-I | TCCTGGGCCCAGCCTGTG |
| RhLl13-O | CAGTCTCTGTGGCTTCCTATG | RhLl13-I | TCCCTCTCCCAGCCTGTGC |
| RhLl14-O | GCTCTGCAGCCTCCTATG | RhLl14-I | TCTCTCTCCCAGCCTGTGCT |
| RhLl15-O | GGCTCTGTGACCTCCTATGAG | RhLl15-I | CAGGTTCTGTGGTTTCTTCTGAGCTG |
| RhLl16-O | GGTTCTTGGACCCATATGAGC | RhLl16-I | GGTTCCGTGGTTTCCTCTGGG |
| RhLl17-O | GGTTCTTGGGCCCAGTATG | RhLl17-I | GGTTCCGTGGTTTCCTCTGAGCTG |
| RhLl18-O | GGTTCTTGGGCCCAGTTTG | RhLl18-I | CAGTCTCTGTGGCCTCCTATGAGCTG |
| RhLl19-O | GGTTCTGTGACCTCCTATGAGC | RhLl19-I | CAGTCTCTGTGGCTTCCTATGAGCTG |
| RhLl20-O | GACTCTGCGGCCTCCTTTG | RhLl20-I | GCTCTGCAGCCTCCTATGAGCTG |
| RhLl21-O | TCTGCGGCCTCCTATGAG | RhLl21-I | GGCTCTGTGACCTCCTATGAGCTG |
| RhLl22-O | TTCCCTCTCGCAGCCTG | RhLl22-I | GGTTCTTGGACCCATATGAGCTG |
| RhLl23-O | GTTCCCTCTCCAAGCCTATG | RhLl23-I | GGTTCTTGGGCCCAGTATGAGCTG |
| RhLl24-O | ATCCTGGGCTCAGGCTG | RhLl24-I | GGTTCTTGGGCCCAGTTTGTGCTG |
| RhLl25-O | GTCCTGGGCCCAGTCTG | RhLl25-I | GGTTCTGTGACCTCCTATGAGCTG |
| RhLl26-O | TCCAATTCCCAGGCTGTG | RhLl26-I | GACTCTGCGGCCTCCTTTGAG |
| RhLl27-O | CAGTGCCAGTGGTCCAGG | RhLl27-I | GGCTCTGCGGCCTCCTATGAG |
| RhLl28-O | GCTCTGTGGCCTCCTATG | RhLl28-I | GGTTCCCTCTCGCAGCCTGTG |
| RhLl29-O | CAGGCTCTGTCATCTCTTGTG | RhLl29-I | GGTTCCCTCTCCAAGCCTATGC |
| RhLl30-O | GGTTCCGTGGTTTCCTCCG | RhLl30-I | CTGGGCTCAGGCTGCC |
| RhLl31-O | GGCTCTGTTGCCTCCTATG | RhLl31-I | TCCTGGGCCCAGTCTGCCC |
| RhLl32-O | GATCCTGGGCTCAGTCTG | RhLl32-I | TCCAATTCCCAGGCTGTGGTG |
| RhLl33-O | GATCCTGGGCTCAGTCTG | RhLl33-I | CCAGTGGTCCAGGCAGGG |
| RhLl34-O | GGTCTTGGGCTGAGGTTG | RhLl34-I | TCCCTCTCCCAGCCTGAGC |
| RhLl35-O | GGGTCCAATTCTCAGGCTG | RhLl35-I | CTCTGTGGCCTCCTATGAGCTG |
| RhLl36-O | GGAGTGGATTCTGAGACTGTG | RhLl36-I | CCTGGGCTCAGTCTGCC |
| RhLl37-O | GTTCCCTCTCCCAGCC | RhLl37-I | GGGTCTTGGGCTGAGGTTGTG |
| RhLl38-O | GTTCCCTCTCACAGCCTG | RhLl38-I | CAGGGTCCAATTCTCAGGCTGTAG |
| RhLl39-O | GTTCCCTCTCCCAGCCTG | RhLl39-I | GGAGTGGATTCTGAGACTGTGGTG |
| RhLl40-O | CAGGTTCCCTCTCTCAGCC | RhLl40-I | GTTCCCTCTCCCAGCCTATGC |
| RhLl41-O | CAGGTTCTCTCTCCCAGTC | RhLl41-I | GGTTCCCTCTCTCAGCCTTTGC |
| RhLl42-O | GTTCCCTCTCCCAGCC | RhLl42-I | GGTTCTCTCTCCCAGTCTGTGC |
| Reversse primer | | Reverse primer | |
| RhLlrev-O | TGCGTGACCTGGCAGCTG | RhLlrev-I | GTAGCTGCTGGCCGCGTACTTG |

# Table S4. Summary of mAb sequences, related to Figure S5.

| Animal | Lineage | ID | HV | HV identity (%) | HJ | H3 | CDR H3 Length | KV | KV identity (%) | KJ | L3 | CDR L3 Length |
|---|---|---|---|---|---|---|---|---|---|---|---|---|
| 08D213 | a | 1A9 | HV4-NL_33*01_S4377 | 92.3 | HJ6-6*01 | ARHSSGFFSLYALDP | 15 | KV1-25*01 | 93.7 | KJ3*01 | QRHDTNPFT | 9 |
| | | ITS112.02 | HV4-NL_33*01_S4377 | 93 | HJ6-6*01 | ARHSSGFFSLYALDP | 15 | KV1-25*01 | 94.4 | KJ3*01 | QRHDTNPFT | 9 |
| | | ITS112.01 | HV4-NL_33*01_S4377 | 94.3 | HJ6-6*01 | ARHSSGFFSLYALDS | 15 | KV1-25*01 | 95.4 | KJ3*01 | QRHDTNPFT | 9 |
| | | ITS112.03 | HV4-NL_33*01_S4377 | 92.3 | HJ6-6*01 | AKHSSGFFSLYALDS | 15 | KV1-25*01 | 95.1 | KJ3*01 | QRHDTNPFT | 9 |
| | | 2D10 | HV4-NL_33*01_S4377 | 94.6 | HJ6-6*01 | ARHSSGYFSLYALDS | 15 | KV1-25*01 | 94.7 | KJ3*01 | QRHDTNPFT | 9 |
| | b | ITS110.01 | HV4-NL_33*01_S4377 | 94 | HJ6-6*01 | ARHSSGWFSLYGLDS | 15 | KV1-25*01 | 95.7 | KJ4*01 | QRHDTTPLT | 9 |
| | | ITS110.04 | HV4-NL_33*01_S4377 | 94 | HJ6-6*01 | ARHSSGWFSLYGLDS | 15 | KV1-25*01 | 94.6 | KJ4*01 | QRHDTTPLT | 9 |
| | | ITS110.02 | HV4-NL_33*01_S4377 | 91.6 | HJ6-6*01 | ARHSSGWFSLYGLDS | 15 | KV1-25*01 | 95.7 | KJ4*01 | QRHDTTPLT | 9 |
| | | ITS110.03 | HV4-NL_33*01_S4377 | 95.7 | HJ6-6*01 | ARHSSGWFSLYGLDL | 15 | KV1-25*01 | 94.3 | KJ4*01 | QRHDTTPLT | 9 |
| | c | 1A11 | HV4-NL_33*01_S4377 | 93.6 | HJ6-6*01 | ARHTSGFFALYGLDS | 15 | KV1-25*01 | 95.8 | KJ3*01 | QQHHTNPFT | 9 |
| | | 2B9 | HV4-NL_33*01_S4377 | 92.3 | HJ6-6*01 | ARHTSGFFALYGLDF | 15 | KV1-25*01 | 95.8 | KJ3*01 | QQHHTNPFT | 9 |
| | | 1D7 | HV4-NL_33*01_S4377 | 92.3 | HJ6-6*01 | ARHTSVFFALYGLDS | 15 | KV1-25*01 | 93.3 | KJ3*01 | QQHHTNPFT | 9 |
| | | 2A1 | HV4-NL_33*01_S4377 | 92.6 | HJ6-6*01 | ARHTSGFFALYGLDS | 15 | KV1-25*01 | 94.7 | KJ3*01 | QQHHTNPFT | 9 |
| | | 1B8 | HV4-NL_33*01_S4377 | 94.3 | HJ6-6*01 | SRHTSGFFALYALDS | 15 | KV1-25*01 | 94.4 | KJ3*01 | QQHHSDPFT | 9 |
| | | 1E5 | HV4-NL_33*01_S4377 | 93.6 | HJ6-6*01 | VRHTSGFFALYGLDS | 15 | KV1-25*01 | 94 | KJ3*01 | QQHHSDPFT | 9 |
| | | 2E8 | HV4-NL_33*01_S4377 | 95 | HJ6-6*01 | SRHTSGFFALKALDS | 15 | KV1-25*01 | 96.8 | KJ3*01 | QQHNTDPFT | 9 |
| | d | 1F11 | HV4-NL_33*01_S4377 | 92.6 | HJ6-6*01 | ARHTSGYFVLYGLDL | 15 | KV1-25*01 | 94.7 | KJ3*01 | QQHYSDPFT | 9 |
| | e | 1C12 | HV4-NL_33*01_S4377 | 92 | HJ6-6*01 | ARHRSGWSWFYYGLDS | 16 | KV1-37*02 | 96.8 | KJ1*01 | QQYNSDPWT | 9 |
| | | 1F3 | HV4-NL_33*01_S4377 | 90.3 | HJ6-6*01 | ARHRSGWSWFHYGLDS | 16 | KV1-37*02 | 96.8 | KJ1*01 | QQYHSDPWT | 9 |
| | | 1E6 | HV4-NL_33*01_S4377 | 95 | HJ6-6*01 | ARHRSGWTWFTYGLDS | 16 | KV1-37*02 | 97.9 | KJ1*01 | QQYNSDPWT | 9 |
| | | 2A9 | HV4-NL_33*01_S4377 | 91.6 | HJ6-6*01 | ARHRSGWTWFYYGLDS | 16 | KV1-37*02 | 98.2 | KJ1*01 | QQYNSDPWT | 9 |
| DBME | a | 1A8 | HV4-NL_33*01_S4377 | 93.6 | HJ6-6*01 | ARHSSGYFTLYGLDS | 15 | KV1-25*01 | 93.3 | KJ3*01 | QYHNSDPFT | 9 |
| | | 2B5 | HV4-NL_33*01_S4377 | 91.6 | HJ6-6*01 | ARHSSGYFTLYGLDS | 15 | KV1-25*01 | 93.3 | KJ3*01 | QYHNSDPFT | 9 |
| | | 2B2 | HV4-NL_33*01_S4377 | 93.3 | HJ6-6*01 | ARHSSGYFTLYGLDS | 15 | KV1-25*01 | 94.4 | KJ3*01 | QYHNSDPFT | 9 |
| | | ITS111.01 | HV4-NL_33*01_S4377 | 93.6 | HJ6-6*01 | ARHSSGYFTLYGLDS | 15 | KV1-25*01 | 94.4 | KJ3*01 | QYHNSDPFT | 9 |
| | | ITS111.02 | HV4-NL_33*01_S4377 | 94.6 | HJ6-6*01 | ARHSSGYFTLYALDS | 15 | KV1-25*01 | 94 | KJ3*01 | QYHNSDPFT | 9 |
| | b | 1A11 | HV4-NL_33*01_S4377 | 85.3 | HJ5-4*03 | ARQEQTLFWVKRFDV | 15 | KV1-37*01, KV1-37*02 | 93.3 | KJ3*01 | QQYYSDPFT | 9 |
| | | 1B2 | HV4-NL_33*01_S4377 | 83.6 | HJ5-4*03 | ARQEQTLFWVKRFDV | 15 | KV1-37*02 | 91.9 | KJ3*01 | QQYYSDPFT | 9 |
| | | ITS114.01 | HV4-NL_33*01_S4377 | 83.6 | HJ5-4*03 | TRQEQTLFWVKRFDV | 15 | KV1-37*02 | 94.7 | KJ3*01 | QQYYSDPFT | 9 |
| | | ITS114.01 | HV4-NL_33*01_S4377 | 86 | HJ5-4*03 | TRQEQTLFWVKRFDV | 15 | KV1-37*02 | 95.1 | KJ3*01 | QQYYSDPFT | 9 |
| | | 1G9 | HV4-NL_33*01_S4377 | 84.9 | HJ5-4*03 | ARQEQTLFWVKRFDV | 15 | KV1-37*02 | 94.7 | KJ3*01 | QQYYSDPFT | 9 |
| | | 2C1 | HV4-NL_33*01_S4377 | 86 | HJ5-4*03 | ARQEQTLFWVKRFDV | 15 | KV1-37*02 | 94.7 | KJ3*01 | QHYYSDPFT | 9 |
| | | 2C9 | HV4-NL_33*01_S4377 | 84.9 | HJ5-4*03 | ARQEQTLFWVKRFDV | 15 | KV1-37*02 | 94 | KJ3*01 | QQYYSDPFT | 9 |
| | | 1G11 | HV4-NL_33*01_S4377 | 86 | HJ5-4*02, HJ5-4*03 | ARQEQTFLWVKKFDV | 15 | KV1-37*02 | 93.7 | KJ3*01 | QQYYGDPFT | 9 |
| | | 2C11 | HV4-NL_33*01_S4377 | 88 | HJ5-4*02, HJ5-4*03 | ARQEQTFLWVKKFDV | 15 | KV1-37*02 | 92.6 | KJ3*01 | QQYYGDPFT | 9 |
| | c | 1B4 | HV4-NL_33*01_S4377 | 89.7 | HJ5-4*03 | TKQDQTLLWVSRFNV | 15 | KV1-37*02 | 94.7 | KJ3*01 | QQYSSDPFT | 9 |
| | | 2D4 | HV4-NL_33*01_S4377 | 89.3 | HJ5-4*03 | TKQDQTLLWVSRFNV | 15 | KV1-37*02 | 95.1 | KJ3*01 | QHYYSDPFT | 9 |
| | | 1E1 | HV4-NL_33*01_S4377 | 89.7 | HJ5-4*03 | TKQDQTLLWVSRFNV | 15 | KV1-37*02 | 95.1 | KJ3*01 | QHYYSDPFT | 9 |
| | | ITS113.01 | HV4-NL_33*01_S4377 | 87.3 | HJ5-4*03 | TRQDQTLLWVSRFTV | 15 | KV1-37*02 | 93.7 | KJ3*01 | QHYYSDPFT | 9 |
| | | ITS113.02 | HV4-NL_33*01_S4377 | 87.3 | HJ5-4*03 | TRQDQTLLWVSRFTV | 15 | KV1-37*02 | 93.3 | KJ3*01 | QQYYSDPFT | 9 |
| | | 1C12 | HV4-NL_33*01_S4377 | 87 | HJ5-4*03 | SRQDQTLWWVSRFTV | 15 | KV1-37*02 | 90.8 | KJ3*01 | HHYYNDPFT | 9 |
| | | 2G4 | HV4-NL_33*01_S4377 | 84.9 | HJ5-4*02, HJ5-4*03 | SRQDQTLWWVSRFTA | 15 | KV1-37*02 | 91.9 | KJ3*01 | LQYFNDPFT | 9 |
| | d | 1A1 | HV4-NL_33*01_S4377 | 86.6 | HJ5-4*03 | ARQDQTMFWVNRFNL | 15 | KV1-37*02 | 96.1 | KJ3*01 | QQYNSDPFT | 9 |
| | | 1B3 | HV4-NL_33*01_S4377 | 87.3 | HJ5-4*03 | ARQDQTMFWVNRFNL | 15 | KV1-37*02 | 96.1 | KJ3*01 | QQYNSDPFT | 9 |
| | | 1C1 | HV4-NL_33*01_S4377 | 88 | HJ5-4*03 | ARQDQTMFWVNRFNL | 15 | KV1-37*02 | 96.5 | KJ3*01 | QQYNSDPFT | 9 |
| | | 1C7 | HV4-NL_33*01_S4377 | 86.3 | HJ5-4*03 | ARQDQTMFWVNRFNL | 15 | KV1-37*02 | 96.8 | KJ3*01 | QQYNSDPFT | 9 |
| | | 1C8 | HV4-NL_33*01_S4377 | 87.6 | HJ5-4*03 | ARQDQTMFWVNRFNL | 15 | KV1-37*02 | 96.8 | KJ3*01 | QQYNSDPFT | 9 |
